# Effect of the Andean Geography and Climate on the Specialized Metabolism of Its Vegetation: The Subtribe Espeletiinae (Asteraceae) as a Case Example

**DOI:** 10.3390/metabo11040220

**Published:** 2021-04-04

**Authors:** Guillermo F. Padilla-González, Mauricio Diazgranados, Fernando B. Da Costa

**Affiliations:** 1 AsterBioChem Research Team, Laboratory of Pharmacognosy, School of Pharmaceutical Sciences of Ribeirão Preto, University of São Paulo, Ribeirão Preto SP 14040-903, Brazil; f.padilla@kew.org; 2 Jodrell Laboratory, Royal Botanic Gardens, Kew, Kew Road, London TW9 3AB, UK; 3 Millennium Seed Bank, Royal Botanic Gardens, Kew, Ardingly, West Sussex, Haywards Heath RH17 6TN, UK; m.diazgranados@kew.org

**Keywords:** andes, espeletia, environment, metabolomics, páramo

## Abstract

The Andean mountains are ‘center stage’ to some of the most spectacular examples of plant diversifications, where geographic isolation and past climatic fluctuations have played a major role. However, the influence of Andean geography and climate as drivers of metabolic variation in Andean plants is poorly elucidated. Here, we studied the influence of those factors on the metabolome of the subtribe Espeletiinae (Asteraceae) using liquid chromatography coupled to high-resolution mass spectrometry data of over two hundred samples from multiple locations. Our results demonstrate that metabolic profiles can discriminate Espeletiinae taxa at different geographic scales, revealing inter- and intraspecific metabolic variations: at the country level, segregation between Colombian and Venezuelan taxa was observed; regionally, between páramo massifs; and locally, between páramo complexes. Metabolic differences in Espeletiinae were mainly explained by geographic isolation, although differences in taxonomic genera, temperature, and elevation, were also important. Furthermore, we found that different species inhabiting the same páramo complex showed stronger chemical similarities than the same species at different locations, corroborating that geographic isolation represents the main driver of metabolic change in Espeletiinae. The current study serves as a starting point to fill in the gaps in how Andean geography and climate have shaped the metabolism of its vegetation and reveal the potential of untargeted metabolomics to study the environmental physiology of plants.

## 1. Introduction

**Figure 1:**
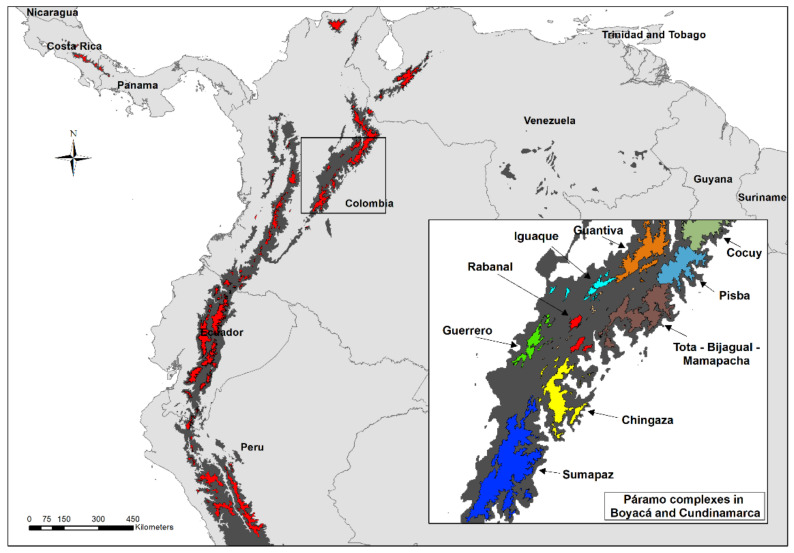
Present-day area covered by páramos (red) and high Andean forests (dark gray). Magnified box shows the páramo complexes within the Boyacá and Cundinamarca massifs labeled in different colors. Map created in the software ArcGIS 10.7.

The tropical Andes are a global biodiversity hotspot considered as one of the most diverse areas on Earth in terms of vascular plant species [1,2]. According to Myers et al. (2000), the Andean mountains represent the most species-rich biodiversity hotspot, with ca. 15% of the world’s plant species within only 1% of the world’s land surface. Andean uplift and past climatic fluctuations are often considered the causal agents for the explosive speciation and adaptive radiation of several Andean plant groups, as these past events provided new habitats and the absence of competition [3,4,5,6,7,8]. With more than 3400 species of vascular plants, a large proportion of this diversity is found in the páramo ecosystem [9,10], a biome regarded as the world’s fastest evolving and coldest biodiversity hotspot [11]. According to Hughes and Eastwood (2006), ‘a high proportion of the 3400 plant species recorded in the northern Andean páramos have evolved in the very recent evolutionary past’ [6]. Páramos represent high elevation Andean grasslands located between ca. 2800 and 4700 m above sea level ‘along the crests of the highest mountain ranges or on isolated mountaintops, like islands in a sea of forest’ [9]. Due to their broken topography and altitudinal restriction, páramos are scattered in the Central and South American tropical mountains of Costa Rica, Panama, Venezuela, Colombia, Ecuador, and northern Peru (Figure 1), and biogeographically function as islands. These ‘sky islands’ allegedly act as barriers promoting allopatric speciation [12].

The distribution of the páramo vegetation depends not only on the topography of the region but also on the Andean climate. With more than 20 known late Pliocene–Pleistocene glaciations during the last 3.5 million years, the past Andean climate was very dynamic [13,14,15,16]. During glacial maxima, the area of páramos was considerably larger than during the interglacial periods, promoting distributional changes of plant communities (both in elevation and latitude) as repeated events of expansion and contraction of the páramo zone took place [17]. The Andean climate, together with the geographic isolation of the páramos, have shaped the evolution and biogeographic history of several plant groups, such as *Lupinus* L. (Fabaceae) [6,8,17,18], *Oreobolus* R. Br. (Cyperaceae) [5,19], *Astragalus* L. (Fabaceae) [20], *Diplostephium* Kunth (Asteraceae) [21], *Loricaria* Wedd. (Asteraceae) [7], *Valeriana* L. (Caprifoliaceae) [22], *Hypericum* L. (Hypericaceae) [23], *Puya* Molina (Bromeliaceae) [24], and Espeletiinae Cuatrec. (Asteraceae) [3,25]. However, the influence of Andean geography and climate as drivers of metabolic variation in Andean plants is poorly understood.

The subtribe Espeletiinae (Asteraceae, Millerieae) represents the most iconic and representative plant group of the páramo ecosystem with ca. 145 species, popularly known as ‘frailejones’ [3,26,27]. The group is divided into eight genera according to morphology: *Carramboa* Cuatrec., *Coespeletia* Cuatrec., *Espeletia* Mutis ex Humb. & Bonpl., *Espeletiopsis* Cuatrec., *Libanothamnus* Ernst, *Paramiflos* Cuatrec., *Ruilopezia* Cuatrec., and *Tamania* Cuatrec. [26,28]. However, serious doubts persist over the taxonomic subdivisions of Espeletiinae, as a recent study proposed that only *Espeletia* should be recognized, with the other seven genera considered as heterotypic synonyms [27]. Species of Espeletiinae are only found in the high Andean forests and páramos of Colombia (*ca*. 90 species), Venezuela (68 species), and northern Ecuador (1 species) [3,26,27]. Based on species distribution patterns, frailejones have two main centers of species diversity and richness (Figure S1): the Venezuelan páramo massif of Mérida (with ca. 50 species) and the Colombian massifs of Boyacá (with 45 species) [3,27,28]. The Colombian páramos of Santander/Norte de Santander (hereafter referred to as ‘Santanderes’ with 41 species) and Cundinamarca (19 species) represent additional centers with lower diversity [3,28,29]. Only 14 species are shared between Colombia and Venezuela, and most are restricted to one or two páramo localities [30]. Considering its high endemicity and strong limitations in long-distance seed dispersal and pollination, the biogeographic history of Espeletiinae has been the focus of several studies over the last years. Based on concatenated analyses including nuclear (internal and external transcribed spacer sequences—ITS and ETS, respectively), plastid (rpl16), and amplified fragment length polymorphism (AFLP) molecular markers, two large clades were recovered: one with primarily Venezuelan species and a second clade with mainly Colombian species (including the shared species with northern Ecuador) [3]. A similar pattern was obtained in a recent phylogenomic analysis based on approximately one million nuclear nucleotides [25], demonstrating that two geographically independent radiations took place in each country (Colombia and Venezuela) and that geographic isolation and past climatic fluctuations have shaped the evolution of this group.

In this context, the subtribe Espeletiinae constitutes a good model to evaluate the effect of Andean climate and geographic isolation on the specialized metabolism of its vegetation. As the specialized metabolism is involved in the interactions that organisms have with their environment [31,32], approaches such as metabolomics, which aims to study the set of metabolites synthesized by organisms, and their expression patterns in response to genetic or environmental influences, may be considered the link between genotype and phenotype [33]. Recent breakthroughs in metabolomics have demonstrated its potential as a powerful tool for understanding different aspects of plant biology, from the chemical interactions occurring between plants and other organisms [34,35] to the response of the plant’s specialized metabolism to environmental stress [31,36] and the characterization of the chemical diversity that mediates plant ecology and evolution [34,35,37]. Metabolomes, like any other phenotypic trait, are influenced by external factors, such as solar radiation, temperature, precipitation, and elevation, under natural conditions. Therefore, metabolomics, in combination with molecular and ecological information, can provide a broader view of the adaptive responses occurring during plant diversification.

Using an ultrahigh-performance liquid chromatography-UV-high-resolution mass spectrometry (UHPLC-UV-HRMS)-based metabolomics approach, we recently showed that Andean geography shaped the metabolome in the genus *Espeletia* [38]. However, in this first study, the contribution of taxonomy and environmental variables (such as solar radiation, seasonality, precipitation, temperature, and elevation) on the metabolic variation of the group was not explored, and it is currently unknown whether Andean geography also affects plant metabolism at different geographic scales and taxonomic ranks. In the present study, we demonstrate that geographic isolation is not only strongly correlated with the metabolic variation of the genus *Espeletia* but also, in broader taxonomic ranks, of the subtribe Espeletiinae and at finer geographic scales than previously thought. We found that while metabolic differences in Espeletiinae were mainly correlated with geography, differences in taxonomic genera, temperature, and elevation, are also significant factors shaping the metabolome of the group.

## 2. Results

### 2.1. Metabolic Fingerprinting and Correlation with Biogeographic Data

**Figure 2:**
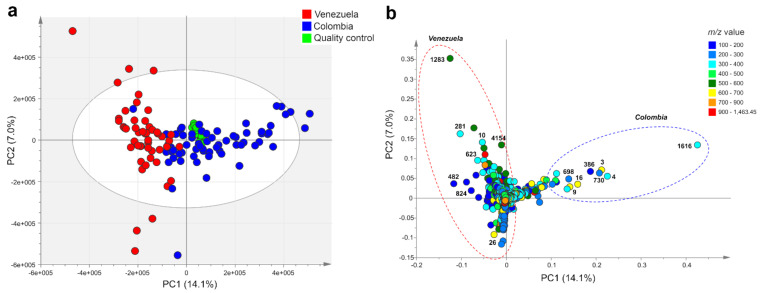
Principal component analysis (PCA) based on metabolic fingerprinting in negative ion mode of 113 taxa of Espeletiinae (representing 210 samples and 4181 variables). (**a**) Scores plot showing the clustering of samples according to their country of origin (Venezuelan samples in red and Colombian in blue) and (**b**) loadings plot. Discriminant mass signals in the loadings plot are labeled according to their MZmine IDs. For compound identities, please refer to Table 1.

Metabolic fingerprinting by UHPLC-UV-HRMS of crude methanol extracts from 210 samples of Espeletiinae detected 4181 mass signals in negative ionization mode. Two types of multivariate analyses were performed with all detected mass features in this mode: unsupervised methods by principal component analysis (PCA) and non-metric multidimensional scaling (NMDS), and a supervised method using decision trees. PCA of the negative mode dataset grouped plant extracts by chemical fingerprint similarity. This analysis revealed the segregation of the samples into two main groups related to the species’ country of origin (Colombia and Venezuela, Figure 2). All Venezuelan samples were grouped in the left quadrant of PC1, while Colombian samples were grouped mainly in the right quadrant, except for one sample (Figure 2a). Analysis of the PCA loadings plot (Figure 2b) revealed that different mass features characterize each group, indicating a differential accumulation of selected metabolites according to the country of origin of each species. The fact that all replicates of the quality control samples clumped together in the PCA scores scatter plot (Figure 2a) suggests good reproducibility both in the extraction process and in the chromatographic analyses.

**Figure 3:**
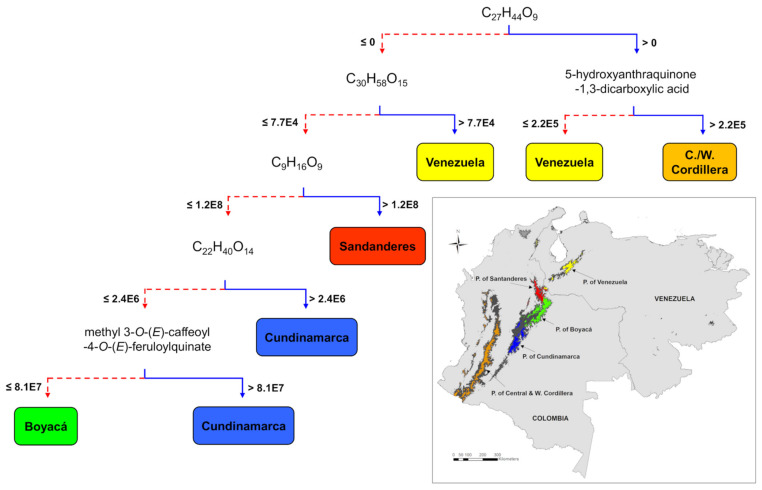
Decision-tree based on metabolic fingerprinting in negative ion mode of 113 taxa of Espeletiinae (representing 210 samples and 4181 variables). J48 tree showing the metabolites (nodes) and their concentration threshold (branches) associated with the geographical origin of species of Espeletiinae. For compound identities, please refer to Table 2. Map created in the software ArcGIS 10.7.

The correlation between the metabolic fingerprints of Espeletiinae and their páramo massifs of origin (Venezuela, Santanderes, Boyacá, Cundinamarca, and Central/Western Cordillera) revealed that species in the same massif tend to be metabolically more similar than geographically distant species (Figure 3). This analysis also showed a high percentage (71.8%) of correctly classified instances of the external validation group. Considering that the decision tree algorithm chooses the variables of the data that most effectively divides its set of samples into subsets enriched in one class [39], the nodes of the tree represent the metabolites (identified by their molecular formula or annotation) that discriminate samples from each geographic locality (Figure 3). As observed in Figure 3, in most cases, the metabolic clustering of species of Espeletiinae reflects the geographic proximity of their respective páramo localities. For example, species located in the páramo massifs of Santanderes, Boyacá, and Cundinamarca, three adjacent locations, clustered nearby based on their similar metabolic fingerprints (Figure 3). However, in one case, groups of species located in distant locations clustered nearby. Species located in the Colombian Central and Western Cordillera were clustered with a group of Venezuelan taxa (Figure 3), despite the large geographic distance between these two páramo massifs (see the discussion section for a plausible explanation). Additionally, the Espeletiinae samples located in Venezuela and Cundinamarca were segregated each in two different subgroups based on the differential accumulation of the metabolites C_27_H_44_O_9_ and C_22_H_40_O_14_, respectively (Figure 3).

**Figure 4:**
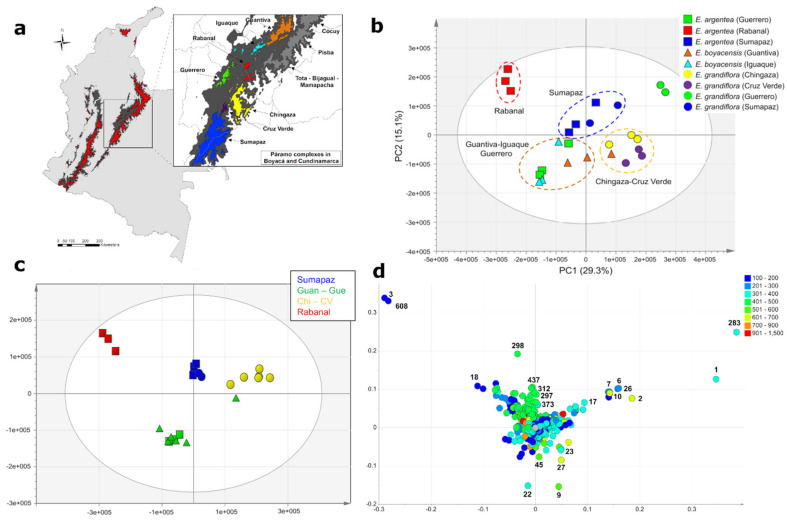
Clustering of 25 individuals from three species of *Espeletia* based on metabolic fingerprinting in negative ion mode (915 variables). (**a**) Map of Colombia with different páramo complexes within the Boyacá and Cundinamarca massifs highlighted in different colors. (**b**) PCA scores plot showing a clustering tendency according to páramo complexes. (**c**) orthogonal partial least squares discriminant analysis (OPLS-DA) (*R*^2^ = 0.96, *Q*^2^ = 0.77) scores plot showing the correlation between metabolic fingerprints and páramo complexes of species from Sumapaz, Guantiva, Iguaque, and Guerrero (Guan–Gue), Chingaza and Cruz Verde (Chi–CV) and Rabanal. Species are represented by different symbols: squares (*E. argentea*), triangles (*E. boyacensis*), and circles (*E. grandiflora*). (**d**) OPLS-DA loadings plot. For compound identities, please refer to Table 3. Map created in the software ArcGIS 10.7.

**Figure 5:**
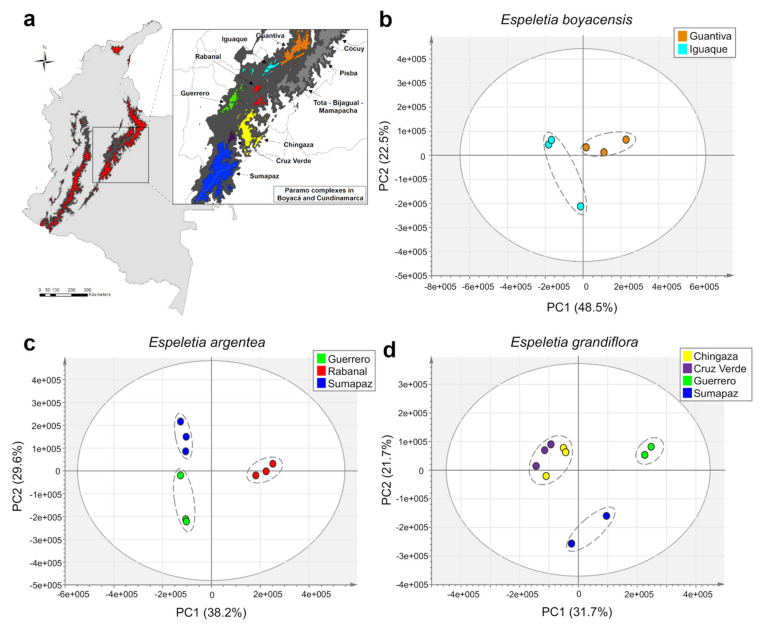
Individual principal component analyses (PCA) based on metabolic fingerprinting in negative ion mode of three species of *Espeletia* (representing 25 samples and 915 variables). (**a**) Map of Colombia with different páramo complexes within the Boyacá and Cundinamarca massifs highlighted in different colors. (**b**) Scores plots showing the metabolic clustering of individuals of *E. boyacensis*, (**c**) *E. argentea,* and (**d**) *E. grandiflora* according to their páramo complex of origin. Map created in the software ArcGIS 10.7.

Further UHPLC-UV-HRMS analysis of 25 individuals belonging to three different species of *Espeletia* (*E. argentea*, *E. boyacensis,* and *E. grandiflora*) (intraspecific dataset) afforded 917 mass signals in the negative ionization mode. PCA of this dataset showed that individual samples tended to cluster according to their respective páramo complex of origin based on their similar metabolic fingerprints (Figure 4). Interestingly, in this analysis, different species inhabiting the same páramo complex showed stronger chemical similarities than the same species at different locations (Figure 4a–c), indicating that geography has a greater influence than taxonomy in shaping the metabolic fingerprints of these species. To confirm this hypothesis, an additional analysis by orthogonal partial least squares discriminant analysis (OPLS-DA) (*R^2^* = 0.96, *Q^2^* = 0.77) was performed with the páramo complexes of origin as the Y variable (Figure 4c). Results from this analysis showed a clear segregation according to the geographical origin of the individual samples. For example, *E. argentea* collected in the páramo of Sumapaz showed closer metabolic similarities with *E. grandiflora* from the same locality than with other individuals of *E. argentea* collected in more distant localities (Figure 4c). The same tendency was observed among individuals of *E. argentea* and *E. boyacensis* collected in the páramos of Guantiva, Iguaque, and Guerrero, three nearby páramo complexes (Figure 4a). Additional analyses by PCA of each species independently (Figure 5) confirmed that a clear intraspecific metabolic differentiation occurs according to the páramo complex of origin of each species. However, in the case of *E. grandiflora*, samples collected in the páramos of Cruz Verde and Sumapaz showed different metabolic fingerprints, even though both páramos belong to the same complex (Figure 5d). In this analysis, individuals of *E. grandiflora* collected in Cruz Verde seem to be metabolically more related to the samples collected in the páramo of Chingaza (Figure 5d).

### 2.2. Accumulation Patterns of Specialized Metabolites in Different Geographic Locations

Analysis of PCA loadings plot (Figure 2b) and the nodes of the decision tree (Figure 3) identified the discriminant metabolites associated with the geographic origin of each group of species. Table 1 shows the discriminant metabolites associated with the country of origin of Colombian and Venezuelan samples of Espeletiinae obtained by the analysis of the PCA loadings plot. According to this analysis, the clustering of Colombian taxa is related to the accumulation of relatively larger amounts of the flavonoids 3-*O*-methylquercetin, quercetin, and a putative biflavonoid with a molecular formula of C_31_H_20_O_14_, among others (Table 1). On the other hand, the clustering of Venezuelan taxa is related to the accumulation of relatively larger amounts of the chlorogenic acids 3,5-di-*O*-(*E*)-dicaffeoylquinic acid, 5-*O*-(*E*)-caffeoylquinic acid, and 1,5-di-*O*-(*E*)-dicaffeoylquinic acid (Table 1), identified by retention time (Rt) and MS comparisons with pure standards (see Supplementary Information). The tentative identification of the other discriminant metabolites (Table 1, Table 2 and Table 3) was performed by interpretation of fragmentation patterns, HRMS and MS^2^ spectra comparisons with compounds previously reported in Espeletiinae or closely related species [38,40,41,42,43,44,45,46,47,48]. Remarkably, differences in the relative abundance of the discriminant metabolites of the species’ country of origin can also be seen in the analysis of raw chromatographic data (Figure S2).

Metabolic differences in species of Espeletiinae related to their páramo massifs of origin (representing their diversification centers) are associated with the differential accumulation of the six metabolites reported in Table 2, identified by the nodes of the decision tree (Figure 3). Interestingly, the decision tree algorithm, which has the advantage of being easily interpreted, usually selects a few variables to segregate samples into subsets effectively. Therefore, in this case, concentration differences in the accumulation of the metabolite with the molecular formula C_27_H_44_O_9_ (Figure 3) effectively segregated the Central/Western Cordilleran taxa and most of the Venezuelan species from the remaining Espeletiinae. However, species from the Central/Western Cordillera of Colombia accumulate relatively higher amounts of 5-hydroxyanthraquinone-1,3-dicarboxylic acid than Venezuelan taxa. The clustering of the species located in Santanderes can be related to the accumulation of higher amounts of a glycosylated metabolite with the molecular formula C_9_H_16_O_9_ when compared to the species from Boyacá and Cundinamarca (Figure 3), while these last two groups of species can be segregated by the differential accumulation of methyl 3-*O*-(*E*)-caffeoyl-4-*O*-(*E*)-feruloylquinate, identified based on MS^2^ data and previous reports for species of the genus *Espeletia* [38,40]. This metabolite was accumulated in relatively higher amounts in species from Cundinamarca.

Analysis of the variable importance in the projection (VIP) scores and OPLS-DA loadings plot of the intraspecific dataset (Figure 4c,d) allowed the recognition of the discriminant metabolites associated with the species’ páramo complex of origin (Table 3). Individuals of *E. argentea* and *E. grandiflora* collected in the páramo of Sumapaz were characterized by the accumulation of five unknown peaks with the molecular formulae C_36_H_38_O (Table 3). Similarly, individuals of *E. argentea* and *E. boyacensis*, collected in the páramos of Guantiva, Iguaque, and Guerrero, accumulated several dicaffeoylquinic acid isomers, such as 1,3-, 1,5-, and 3,4-di-*O*-(*E*)-dicaffeoylquinic acid, in addition to chlorogenic acid and 2,3,5, or 2,4,5-tricaffeoylaltraric acid, all identified based on Rt and MS^2^ comparisons with pure standards (see Supplementary Materials). On the other hand, individuals of *E. grandiflora* located in the páramos of Chingaza and Cruz Verde accumulated relatively higher amounts of several flavonoids, including quercetin, 3-*O*-methylquercetin, and a putative biflavonoid with the molecular formula C_31_H_20_O_14_, in addition to altraric acid and protocatechuic acid (Table 3). Lastly, individuals of *E. argentea* collected in the páramo of Rabanal were segregated by the presence of gluconic acid and a putative hexose (Table 3). Detailed information, including common names, retention time, *m/z* values, MS^2^ fragment ions (AIF mode), and confidence level achieved in the identification of all discriminant metabolites, is summarized in Table S1. Interpretation of fragmentation patterns is discussed in the Supplementary Materials.

### 2.3. Effects of Geography, Taxonomy, and Climate on Metabolic Variation

**Figure 6:**
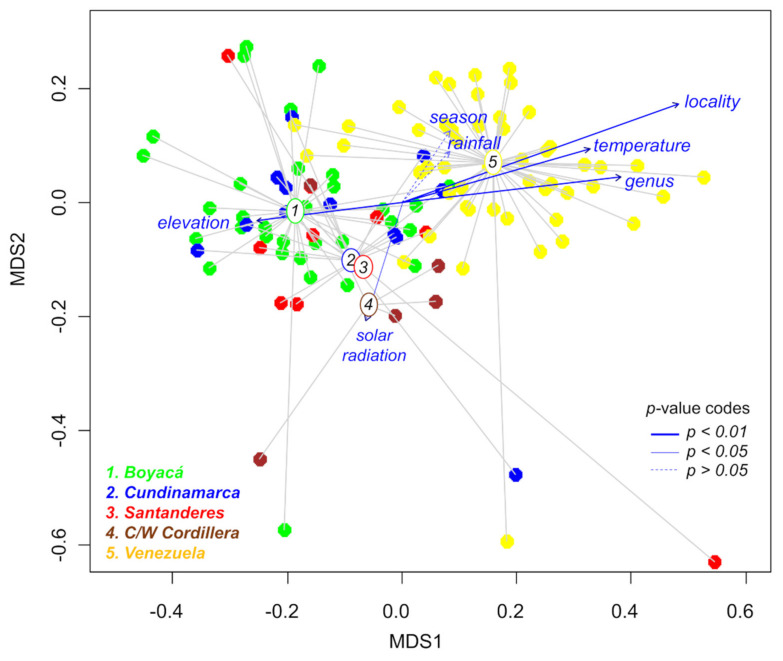
Non-metric multidimensional scaling (NMDS) showing the correlation between the metabolic fingerprints (represented by 4181 variables) of 109 taxa of Espeletiinae obtained by ultrahigh-performance liquid chromatography-high-resolution mass spectrometry (UHPLC-HRMS) in negative ion mode and their geographic localities, taxonomic genus, elevation, and environmental variables.

The concatenated analysis by NMDS of metabolomic data and external variables allowed testing whether the locality of collection, elevation, taxonomic genus, temperature, precipitation, solar radiation, and seasonality (Table S2) have statistically significant effects on the metabolic fingerprints of Espeletiinae (Figure 6). This analysis (stress value = 0.11 and 0.04 for the metabolomic and environmental datasets, respectively) revealed that geography constitutes the main driver of metabolic change in Espeletiinae (Table 4), accounting for 53% of the metabolic variation. The taxonomic genus, elevation, and temperature also had a significant correlation (*p* < 0.01) with the metabolomic dataset, but with lower correlation coefficients (Table 4), while solar radiation showed a lower correlation but still significant (*p* < 0.05). The variables precipitation and seasonality had no significant influence (Table 4). Additional NMDS analyses considering the Colombian and Venezuelan samples independently (Figure S3) showed that temperature and elevation are statistically correlated (*p* < 0.01) only with the Venezuelan group, while the genus and location had a significant correlation with both Colombian and Venezuelan samples.

## 3. Discussion

### 3.1. Metabolomic-Biogeographic Patterns Resemble Phylogenetic Hypotheses in Espeletiinae

Although Andean topography and past climatic fluctuations have played a major role in the biogeographic history of the páramo vegetation [3,5,6,7,8,17,18,19,20,21,22,23,24,25], the influence of those factors as drivers of metabolic variation in Andean plants is still poorly understood. A recent study based on UHPLC-UV-HRMS concluded that the metabolome of the genus *Espeletia* is strongly correlated with the geographic origin of its species [38], but the influence of taxonomy and climate on the metabolic variation of the group was not explored. In the present study, we found that geographic isolation is not only correlated with the metabolic variation of the genus *Espeletia* but also, in broader taxonomic ranks, of the subtribe Espeletiinae and at finer geographic scales than previously thought. These metabolomic–biogeographic patterns agree with and elaborate upon previous results obtained using molecular markers [3,25]. On a country level, we found a major split between Colombian and Venezuelan taxa with significant differences in the accumulation of flavonoids and chlorogenic acids in species from each country, respectively (Table 1). This pattern, previously described only for the genus *Espeletia* [38], remains constant as the number of samples of the same genus increases and even when species from other genera, such as *Espeletiopsis*, *Libanothamnus*, *Ruilopezia,* and *Paramiflos,* are included. Although it is still unknown whether this country-level metabolomic segregation applies to all Espeletiinae species, this pattern is consistent with the molecular phylogenies of the group based on single molecular markers (ITS, ETS, rpl16, and AFLPs) [3] and genomic evidence [25].

The clustering pattern observed at the regional and local levels agrees with recent studies and previous hypotheses about the geographic diversification of the subtribe [3,28]. According to Pouchon et al. (2018), the common ancestor of Espeletiinae appeared in today’s border between Colombia and Venezuela, and two rather independent radiations took place in each country, while dispersal events across the Táchira depression, at the border of both countries, never involved lineages belonging to the genus *Espeletia* or *Espeletiopsis* [25]. The Táchira depression isolates the Colombian Andes from the Venezuelans. For instance, recent evidence suggests that even during the coldest Pleistocene glaciations, there was no continuous connectivity between core areas in the Colombian Eastern Cordillera and the Venezuelan Cordillera de Mérida [13,50]. In Colombia, the Espeletiinae radiation followed mainly southward migrations along the Eastern Cordillera, where frailejones established themselves and diversified into three main páramo massifs—Santanderes, Boyacá, and Cundinamarca to subsequently reach the páramos of the Colombian Central and Western Cordillera (Figure 3) and those of northern Ecuador [3,28]. Our results suggest that species within these three páramo massifs are metabolically more related to each other than to geographically distant species (Figure 3). According to Flantua et al. (2019), although the distribution of Colombian páramos changed substantially between periods of connectivity and fragmentation, even during the periods of highest fragmentation, two large páramo islands remained constant in the Colombian Eastern Cordillera, surrounded by smaller ‘satellite islands’ [13]. These two large ‘sky islands’ represent the current páramos of Santanderes (i.e., Santander/Norte de Santander) and Boyacá on one island, and Cundinamarca on the other [13]. In contrast, páramos in the Venezuelan Cordillera de Mérida seem to have been restricted to a single core area during interglacial periods [13], which could help explain the metabolomic differences among species in those distinct páramo massifs.

At the local level, our metabolomic analyses of 25 individuals from three species (*E. argentea*, *E. boyacensis,* and *E. grandiflora*) demonstrated that the metabolomic–biogeographic clustering occurs at smaller geographic and taxonomic scales than previously reported [38]. Furthermore, we observed an interesting pattern in which different species inhabiting the same páramo complex are metabolically more related to each other than individuals of the same species collected on different sky islands (Figure 4). This result suggests that geography has a greater influence than taxonomy in shaping the metabolic fingerprints of *Espeletia*. However, the reason why populations of *E. grandiflora* collected in Cruz Verde showed greater metabolic similarity within populations from Chingaza than with those of the páramo of Sumapaz remains unknown, considering that Cruz Verde and Sumapaz belong to the same páramo complex (Figure 5a). Population genetic studies of *E. grandiflora* could potentially help clarify this phenomenon in the future. According to Diazgranados and Barber (2017), ‘expansions (with reconnection) and contractions (with isolation) of the páramo ecosystem during the Pleistocene glaciations and interglaciations, respectively, could have played a major role in the radiation and dispersion of these taxa’ at different geographic scales in the Eastern Cordillera of Colombia [3]. It is likely that the metabolic clustering of the frailejones represents the most recent vicariance events that occurred after the last glacial when populations of Espeletiinae were already established along the Colombian Andes. Therefore, our results, together with previous studies, suggest that geographic isolation not only favored speciation mechanisms [3,25] but also the accumulation of metabolic differences in species growing in different localities. However, detailed studies on the timing of chemical evolution in a well-resolved and complete phylogeny will be required to answer this properly.

### 3.2. Combined Effects of Geography, Taxonomy, Phylogeny, and Climate on Plant Metabolic Variation

Although we cannot rule out that other factors, such as soil composition and/or microclimate, could be acting independently in each locality shaping the metabolomes of Espeletiinae, geographic isolation seems to play a dominant role. However, as previously mentioned, the taxonomic genus, temperature, and elevation (Table 4) are also significant factors shaping the metabolome of the group, suggesting there is a combined effect of geography, taxonomy, and climate on metabolic variation. A similar observation was recently put forward in a more comprehensive study including 416 species of Alpine plants [51]. According to the authors, phytochemical diversity and identity can be predicted in the landscape as a function of phylogenetic information, as well as of climatic, topographic, and edaphic factors [51]. Similarly, phylogenetic variation and environmental factors, including climate and soil parameters, had a significant influence on the alkaloid composition of *Cinchona calisaya* Wedd. (Rubiaceae) [52]. In our study, we found that accentuated differences in the accumulation of specific metabolites from different chemical classes, including flavonoids, chlorogenic acids, and sugars, are preferentially accumulated at lower geographic scales (Table 2 and Table 3). From an environmental metabolomics perspective, previous studies with *Smallanthus sonchifolius* (Poepp. and Endl.) H. Robinson (Asteraceae), a species belonging to the sister genus of Espeletiinae, have demonstrated that higher amounts of flavonoids and chlorogenic acids are accumulated in response to increased levels in solar radiation and temperature [36], which represent two statistically significant factors accounting for the metabolic variation seen between Colombian and Venezuelan taxa. According to Cortés et al. (2018), geographical isolation and the mountains’ environmental variation at a local scale played both an important role in the diversification of *Espeletia* [53], illustrating the importance of environmental variation in generating multiple adaptations in a fast-evolving ecosystem, such as the páramo.

Although chemical convergence could help explain the cases in which species located in distant places clumped together, such as the Colombian Central/Western Cordillera and Venezuelan taxa seen in the decision tree (Figure 3), this result must be considered with caution as analysis of the same dataset by a different algorithm (NMDS, Figure 6) clearly differentiated between both groups. An alternative explanation implies that those geographically distant groups/taxa could be sharing ‘ancestral’ metabolites, which have remained unchanged over time. The explosive radiations within the Espeletiinae have likely happened repeatedly under the influence of glaciations and interglaciations along the geographic range, suggesting that recent species might have appeared even nearby the group’s center of origin. Further analysis on the timing of chemical evolution in the Espeletiinae would be required to answer this properly.

### 3.3. Perspectives of Metabolic Variation in the Context of Plant Diversification

The similarities found between the metabolic differentiation of Espeletiinae and its molecular phylogeny suggest that specialized metabolites could be involved in the adaptive responses occurring during plant diversification. Previous studies have demonstrated there is a strong adaptive component in the diversification and biogeographic history of several Andean groups, such as *Lupinus* [17,54] and Espeletiinae [25]. According to Nevado et al. (2016), the extremely high diversification rates seen in Andean members of the genus *Lupinus* are associated with increased rates of positive selection acting on genome-wide coding and regulatory regions [54]. Thus, ‘while geographical isolation may initiate speciation, species differentiation in the face of changing environmental conditions is only achieved once positive selection drives fixation of different adaptive alleles in each species’ [54]. The present study opens an intriguing new perspective of research, as some of those adaptive alleles could be related to the biosynthesis of specialized metabolites serving important ecological functions related to plant adaptation.

## 4. Materials and Methods

### 4.1. Plant Material

Young leaves from 210 samples of Espeletiinae Cuatrec. Including members from five morphological genera (i.e., *Espeletia*, *Espeletiopsis*, *Libanothamnus*, *Ruilopezia*, and *Paramiflos*) were collected during major botanical expeditions between 2007 and 2015 in ca. 70 páramo locations in Colombia and Venezuela (Table S2). Each geographic locality was sampled multiple times in different seasons over the course of eight years (Table S2). Samples were collected and immediately deposited inside resealable zipper storage bags containing silica gel beads with a humidity indicator. Samples contaminated with dirt or fungus were excluded from the extraction process. Taxonomical identification resulted in 168 samples classified to the rank of species (including replicates) and 42 samples classified to the rank of genus due to their lack of conclusive diagnostic characters, totaling 113 individual taxa (Table 5). Complete information about the taxonomic identity, geographic locality, elevation, and environmental variables associated with each collection is reported in Table S2. The same samples used in this study were also used in previous molecular studies to reconstruct the phylogeny of the group [3]. Vouchers of the Colombian species were deposited at the COL, ANDES, FMB, and JBB herbaria, and the Venezuelan species were deposited at the MER herbarium according to the collection numbers reported in Table S2. Collections were made under permits No. 2698 of 09/23/2009 and No. 2 of 02/03/2010 (Ministerio de Ambiente, Colombia) and IE-126 (Venezuela, authorized by Dr. Petr Sklenář).

### 4.2. Environmental Variables

Climatological data available in the WorldClim database (http://www.worldclim.org/, accessed on 1 October 2020) was used to investigate the potential influence of seasonality and other environmental variables on the metabolic fingerprints of Espeletiinae. Average monthly values of temperature (°C), solar radiation (kJ/m^2^/day), and precipitation (mm) were extracted for each collection site (as GPS coordinates) considering a spatial resolution of 30 s (~1 km^2^). The collection date was then used to assign the corresponding values of temperature, solar radiation, and precipitation to each sample (Table S2). To assign the most likely season of collection, we built a histogram for each collection locality with the multiannual monthly precipitation averages. With the resulting histograms containing the seasonality of climate in the páramos (classified as unimodal, bimodal, trimodal, and tetramodal) [55], we assigned the collection season to each sample based on their collection date (Table S2). Seasons were coded as ‘dry season’, ‘rainy season’, ‘rainy season to dry season’, and ‘dry season to rainy season’ following Rangel-Ch [55]. 

### 4.3. Sample Preparation

Two independent experiments were carried out to correlate the metabolome of Espeletiinae with its biogeography. The first experiment focused on the interspecific chemical variability displayed by species collected in five different páramo massifs representing the main centers of biogeographic diversification of the subtribe Espeletiinae in Colombia and Venezuela [28]: the Venezuelan massif of Merida, and the páramo massifs of Santander/Norte de Santander, Boyacá, Cundinamarca, and Central/Western Cordillera in Colombia. Thus, individuals from the same species collected in the same páramo massif (biological replicates) were pooled into a single sample before grinding and extraction (see below). In this experiment, the 42 samples classified to the rank of genus were considered as different taxa to avoid mixing potentially different species.

**Figure 7:**
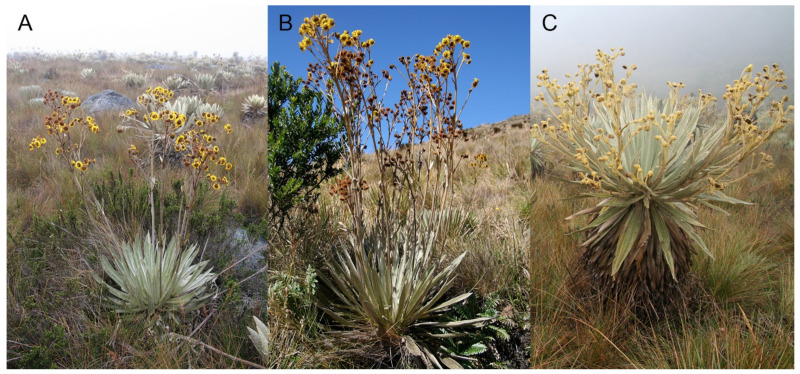
Representative individuals of (**A**) *Espeletia argentea* Humb. & Bonpl., (**B**) *E. boyacensis* Cuatrec., and (**C**) *E. grandiflora* Humb. & Bonpl.

The second experiment focused on the intraspecific chemical variability displayed by individuals of the same species collected in different páramo complexes within the same massif (lower geographic scale). In this analysis, 25 individuals from three species (*Espeletia argentea* Humb. & Bonpl., *E. boyacensis* Cuatrec., and *E. grandiflora* Humb. & Bonpl., Figure 7) with a wide geographic distribution were extracted and analyzed independently (Table S3). In this case, individual samples were collected in seven different páramo localities, five of them located in the massif of Cundinamarca (páramos of Sumapaz, Chingaza, Cruz Verde, Guerrero, and Rabanal) and two in the massif of Boyacá (páramos of Iguaque and Guantiva), all in Colombia. Three biological replicates were analyzed for each species in each locality.

The extraction process used in this study was based on the protocol of extraction and analysis of plant tissues for metabolomic studies by UHPLC-UV-HRMS (Thermo Scientific, Waltham, MA, USA) reported by De Vos et al. [56] with some modifications. Plant leaves (20 ± 0.5 mg) were ground with liquid nitrogen in a pestle and mortar and transferred to Eppendorf tubes where 2 mL of a 7:3 *v*/*v* MeOH-H_2_O mixture (LC-MS and Milli-Q grade, respectively) were added [56]. After the addition of the extraction solvent, tubes were placed in an ultrasonic bath for 15 min at 25 °C using a frequency of 40 kHz. Samples were subsequently centrifuged at 19,975× *g* for 10 min at room temperature, and the supernatant was then partitioned with 0.5 mL of n-hexane (HPLC grade) to eliminate fats and pigments and then filtered through a 0.2 µm PTFE filter. To avoid the possibility of degradation after solvent extraction, samples were extracted (in batches of ten) right before chromatographic analyses, and no more than a few hours passed after extraction and mass spectrometry detection. In this time frame, samples were always kept in the UHPLC autosampler at a constant temperature of 10 °C. Samples were randomly analyzed by arbitrarily selecting tubes to minimize statistical bias.

To check for the reproducibility of the extraction process, 10 mg of each of the 210 samples were mixed and placed into three different 50 mL falcon tubes where the extraction process previously described was carried out while keeping the same ratio of plant material and solvent. These three samples represent three independent analytical replicates (pool1, pool2, and pool3), which were separately and randomly analyzed in the UHPLC-UV-HRMS for quality control purposes.

### 4.4. UHPLC-UV-HRMS Analysis

The UHPLC-UV-HRMS experiments were performed on an Accela UHPLC (Thermo Scientific) apparatus with an 80 Hz photodiode array detector (PDA) coupled to an ESI-Orbitrap mass spectrometer Exactive Plus (Thermo Scientific).

Chromatographic separation of plant extracts (4 μL) was performed using a core shell C18 column (Kinetex 1.7 μm XB, 150 × 2.1 mm, Phenomenex, Torrance, CA, USA) connected to a C18 guard cartridge (Security GuardTM Ultra cartridge, Phenomenex). The mobile phase consisted of purified H_2_O with 0.1% formic acid (line A) and MeCN with 0.1% formic acid (line B). The separation was performed at a flow rate of 400 μL.min^−1^. The elution gradient was 0–15 min, 5–50% B; 15–20 min, 50% B; 20–30 min, 50–100% B; 30–35 min (column washing), 100% B; 35–40 min (column equilibration), 100–5% B. The oven temperature was set at 45 °C, while the auto-injector temperature was kept at 10 °C. The PDA detector was set to record between 200 and 600 nm.

The column effluent was analyzed by ESI-HRMS (resolution of 70,000 FWHM) and ESI-HCD MS/MS (resolution of 35,000 FWHM) in both positive and negative ionization modes using the Full scan and AIF modes. The mass spectra were acquired and processed using the software Xcalibur (Thermo Scientific). Total ion current (TIC) chromatograms were recorded between 100 and 1500 *m/z*. The spray voltage was programmed to +3.6 kV and −3.2 kV for each ionization mode. The capillary and heater temperatures were set at 320 and 300 °C, respectively. Additional parameters for the mass spectrometer included: automatic gain control (AGC) target, 1.0 × 10^6^; maximum injection time, 100 ms; sheath gas flow rate, 30; auxiliary gas flow rate, 10; sweep gas flow rate, 11; S-lens RF level, 50; and HCD, normalized collision energy (NCE), 35 eV. N_2_ was used as the drying, nebulizer, and fragmentation gas.

All plant extracts were randomly analyzed, and each of the three analytical replicates (pool1, pool2, and pool3) were injected five times along the chromatographic sequence (quality control samples) to check for reproducibility in the extraction process and chromatographic analysis.

### 4.5. Data Preprocessing

Each set of chromatographic raw data was sliced into two sets according to the ionization mode (positive and negative) and transformed into mzXML format using the MSConvert package from the software ProteoWizard 3.0.9798 (ProteoWizard Software Foundation, USA). Peaks detected in the last 5 min of the chromatographic run (equilibration stage) were excluded from the analyses. Considering that the subtribe Espeletiinae is especially rich in phenolic compounds [40,57,58], which also represents the main class of metabolites correlated with the biogeographic segregation of *Espeletia* [38], the negative ionization mode was selected for further preprocessing steps. These data were processed by MZmine 2.21 [59] to perform peak detection, peak filtering, chromatogram construction, chromatogram deconvolution, isotopic peak grouping, chromatogram alignment, gap filling, duplicate peaks filter, fragment search, and the search for adducts and peak identities using an in-house chemical structure database (see below). The following key MZmine parameters were used for data processing: exact mass for mass detection considering a noise level at 1.0 × 10^6^; Lorentzian function for the peak shape considering a resolution of 70,000; minimum peak height at 5.0 × 10^6^; *m/z* tolerance at 0.002 or 5.0 ppm; retention time tolerance of 0.7 min; XCMS algorithm for chromatogram deconvolution; RANSAC method for chromatogram alignment; and duplicate peaks filter as filtering algorithm.

After raw data preprocessing in MZmine, the acquired data were exported in csv format and edited as Excel spreadsheets (Microsoft Windows 10, Redmond, WA, USA), where peaks detected in the blank sample (extraction solvent) were subtracted from the peaks detected in the samples to eliminate possible interfering variables. A data matrix containing the species (as rows) versus their peak areas (as columns) was built for the multivariate analyses. Final matrices and raw chromatographic data supporting our results are available in the MetaboLights (Identifiers: MTBLS943 and MTBLS944) and MassIVE (identifier MSV000085122) repositories.

### 4.6. Multivariate Analyses and Correlation with Biogeographic and Environmental Variables

Unsupervised and supervised multivariate statistical analyses and machine learning algorithms were performed in the software R 3.0.3 (R Foundation for Statistical Computing, Austria), Weka 3.6 (University of Waikato, New Zealand), and SIMCA P 13.0.3.0 (Umetrics, Sweden). Initially, PCA was performed as an exploratory method to determine clustering trends based on overall similarity in the sample’s metabolic fingerprints. Before this analysis, the variables were scaled by the method of Pareto. This scaling method was chosen because it reduces the relative importance of highly concentrated metabolites while preserving the structure of the data partially intact [60,61,62]. In Pareto scaling, variables stay closer to the original measurement, avoiding the data becoming dimensionless and the inflation of noisy peaks, as observed in other methods, such as autoscaling and log transformation [60]. A supervised analysis by decision trees was used as a classification algorithm to test whether species from the same páramo massif share specific metabolic traits (regional-level segregation). This method assumes a non-linear relationship between variables and can more effectively describe relationships in complex datasets, in which linear methods (such as PLS-DA) show a poor performance [51]. Decision trees were built using the unscaled data in accordance with [63]. This analysis was performed using the C4.5 algorithm (J48 in Weka 3.6), which uses a top-down strategy splitting data at the top level in two. It then works on each half, further splitting in a top-down manner to produce an optimal tree that can separate the classes [51]. We used the entropy function as splitting criterion and the following parameters: Classifier = Trees—J48, BinarySplits = False, ConfidenceFactor = 0.25, Debug = False, MinNumObj = 5, NumFolds = 3, ReducedErrorPruning = False, SaveInstanceData = False, SubtreeRaising = True, Unpruned = False, and UseLaplace = False. To avoid overfitting, a pruning strategy was adopted by configuring MinNumObj to 5, which prevents the software from continuing splitting if the nodes become very small [39]. In this analysis, 66% of the observations were used to build the model, while the remaining 33% were considered as an external validation set. As a complementary validation strategy, repeated training and testing were performed by changing the random seed value from 1 to 10. In all cases, the generated models had a similar performance of correctly classified instances (average = 63%) from the external validation group. To test for qualitative differences in metabolic fingerprints, chromatographic peak areas were transformed to binary values, and the resulting matrix was analyzed by PCA (Figure S3) and decision trees (Figure S4) following the same parameters previously described. Experimental results showed a certain level of correlation between binary metabolic patterns and biogeographic data, especially at the country level (Figures S3 and S4). However, at lower geographic scales (páramo massifs), the binary matrix showed an inferior correlation compared to the original matrix, demonstrating that quantitative metabolic variations are more informative than qualitative differences in the correlation between metabolomic data and biogeographic information in Espeletiinae. Thus, the original matrix was used for further analyses. Model statistics for each dataset are reported in Table S4.

To explore the influence of environmental variables (temperature, solar radiation, precipitation, and seasonality), elevation, taxonomic genus, and geographic locality on the metabolic variation of Espeletiinae, we ran an NMDS analysis in the R package vegan using the Bray–Curtis dissimilarity index. The envfit function was used to obtain the *p*-value of correlation between the environmental variables and the metabolomic dataset considering 10,000 permutations. The logarithmic scale was applied to the environmental dataset before the NMDS analysis in accordance with previous reports [31,36]. Four taxa (#2,15,24, and 46, Table S2), whose biological replicates were collected in different seasons, were excluded from this analysis. This non-metric approach is especially suited when a linear relationship between the variables is not expected, such as the concentration of metabolites and its relationship with changes in, e.g., elevation gradients, temperature, solar radiation, or taxonomic genera. The Bray–Curtis index quantifies the compositional dissimilarity between different sites making it especially relevant to the purpose of our study.

### 4.7. Selection and Annotation of Discriminant Metabolites

The discriminant variables of the geographical origin were obtained by the analysis of the PCA and OPLS-DA loadings plots and the nodes of the decision tree. To select the discriminating variables accounting for the observed groups in the PCA of the interspecific dataset, we chose the first eight to ten variables with a contribution higher than 0.7 in PC1 and PC2 in the loadings plot. For the intraspecific dataset, a supervised method by OPLS-DA was performed, considering the PCA groups as the Y variable. Two different criteria were applied to extract the discriminant variables: a variable importance in the projection value greater than 1.0 and their contribution in the loadings plot (first variables with a value higher than 0.7). The performance of the OPLS-DA model was validated by two different methods. In the first method, the data were randomly divided into two groups. Seventy percent of the observations (79 samples) were used as a training set, and the remaining 30% (34 samples) as an external validation set. In the second method, we performed ten raw permutations to check the stability of the Q^2^ value [64]. Both methods showed consistent results, validating our results. Discriminant variables indicated by the multivariate statistical analyses were tentatively annotated by comparing their accurate mass measurements, UV spectra, fragmentation patterns, and database searches. Compounds were reported with varying levels of confidence according to the four levels reported in the Metabolomics Standards Initiative (MSI) [49]. Level 1 (identified compounds) corresponds to the metabolites identified by co- characterization (Rt and accurate MS) with authentic standards analyzed under identical experimental conditions. These standards were donated or previously isolated and characterized in our laboratory and are available in our library of pure compounds. Metabolites reported in level 2 (putatively annotated compounds) were characterized upon accurate MS comparisons with compounds reported in the literature, in the Dictionary of Natural Products (DNP, http://dnp.chemnetbase.com, accessed on 15 July 2020) or our in-house chemical structure database of the Asteraceae family (AsterDB, www.asterbiochem.org/asterdb, accessed on 15 July 2020) and by interpretation of fragmentation patterns. In level 3 (putatively characterized compound classes), the chemical class of metabolites was tentatively proposed by comparison of accurate MS values with online databases and chemotaxonomy information, while compounds reported in level 4 correspond to unknown metabolites with only their respective molecular formula.

## 5. Conclusions

Based on comprehensive metabolomic studies combined with statistical methods and machine learning algorithms, we described the influence of Andean geography and climate as drivers of metabolic variation in the subtribe Espeletiinae. Our results suggest that although environmental factors, such as temperature, elevation, and solar radiation contribute to the metabolic variation in Espeletiinae, geography is the most significant factor shaping the metabolome of the subtribe, followed by taxonomy. Considering that metabolic differences were mainly related to quantitative composition yields, rather than qualitative production of distinct metabolites, such differences could be attributed to transcriptional or posttranscriptional regulations (e.g., differential levels of gene expression by geographic locality) rather than composition changes in genes at the genomic level. Therefore, our results suggest that putative changes in gene expression levels could play an important role in the adaptation and diversification of Espeletiinae lineages, as previously reported for Andean lineages on the genus *Lupinus* [54]. However, transcriptomic studies are still necessary for a deeper understanding of gene expression patterns in this remarkable case of rapid plant diversifications on sky islands.

## Figures and Tables

**Table 1:** Discriminant metabolites of the country of origin of 113 taxa of Espeletiinae selected based on the analysis of the PCA loadings plot (Figure 2b). Metabolites sorted by their added contribution in the first two principal components. See Supplementary Information for compounds annotation.

ID	*m/z*	Rt min	Identity or Molecular Formula	Confidence Level *
**Colombia**				
1616	315.051	11.1	3-*O*-methylquercetin	1
4	301.035	10.7	quercetin	1
3	631.109	11.1	dimer of 3-*O*-methylquercetin	1
730	299.020	15.0	biflavonoid (C_31_H_20_O_14_) fragment	3
386	153.018	2.8	protocatechuic acid	1
16	615.079	15.0	biflavonoid (C_31_H_20_O_14_)	3
9	695.124	9.7	2,3,5 or 2,4,5-tricaffeoylaltraric acid	1
698	299.020	11.1	3-*O*-methylquercetin fragment	1
2217	301.072	13.6	C_16_H_14_O_6_	4
484	209.029	0.9	altraric acid	2
**Venezuela**				
1283	515.119	8.4	3,5-di-*O*-(*E*)-dicaffeoylquinic acid	1
281	353.088	4.5	5-*O*-(*E*)-caffeoylquinic acid	1
10	329.067	14.5	di-*O*-methylquercetin	2
4154	515.119	8.1	1,5-di-*O*-(*E*)-dicaffeoylquinic acid	1
623	317.212	22.0	hydroxy-*ent*-kauren-18-oic acid	3
482	191.055	1.0	quinic acid	1
824	171.066	7.6	C_8_H_12_O_4_	4
26	609.125	9.9	quercetin-3-*O*-cinnamoyl-hexoside	2

* Confidence level achieved in the identification of metabolites: 1 (high), identified by retention time (Rt) and accurate MS comparisons with a reference standard; 2 (intermediate), identified by accurate MS comparisons and online searches in the Dictionary of Natural Products and AsterBioChem databases and by interpretation of fragmentation patterns; 3 (low), chemical class suggested by accurate MS comparisons with online databases and chemotaxonomy information; 4 (lowest), unknown metabolites [49]. Discriminant metabolites were selected based on their contribution in the loadings plot (≥0.7 in PC1 and PC2).

**Table 2:** Discriminant metabolites of the páramo massif of origin of 113 taxa of Espeletiinae selected as nodes in the decision tree (Figure 3). Refer to Figure 3 for geographic localities and concentration threshold associated with each metabolite ID. See Supplementary Information for compounds annotation.

ID	*m/z*	Rt	Identity or Molecular Formula	Confidence Level *
1208	511.292	12.9	C_27_H_44_O_9_	4
3047	311.020	12.6	5-hydroxyanthraquinone-1,3-dicarboxylic acid	3
3560	657.292	10.6	C_30_H_58_O_15_	4
109	267.072	1.0	glycosylated metabolite (C_9_H_16_O_9_)	3
4724	527.235	4.2	C_22_H_40_O_14_	4
2591	543.151	8.0	methyl 3-*O*-(*E*)-caffeoyl-4-*O*-(*E*)-feruloylquinate	2

* Confidence level achieved in the identification of metabolites: 1 (high), identified by Rt and accurate MS comparisons with a reference standard; 2 (intermediate), identified by accurate MS comparisons and online searches in the Dictionary of Natural Products and AsterBioChem databases and by interpretation of fragmentation patterns; 3 (low), chemical class suggested by accurate MS comparisons with online databases and chemotaxonomy information; 4 (lowest), unknown metabolites [49].

**Table 3:** Discriminant metabolites of the páramo complex of origin of 25 individuals of *Espeletia* selected by the analysis of the orthogonal partial least squares discriminant analysis (OPLS-DA) loadings plot and variable importance in the projection (VIP) scores (Figure 4d). Metabolites are sorted by their VIP score. See Supplementary Materials for compounds annotation.

ID	*m/z*	Rt	Identity or Molecular Formula	VIP Score	Confidence Level *
	**Sumapaz**				
298	485.283	35.4	C_36_H_38_O	3.26	4
437	485.283	34.2	C_36_H_38_O	1.92	4
373	485.283	28.1	C_36_H_38_O	1.58	4
312	485.283	32.5	C_36_H_38_O	1.57	4
297	485.283	31.5	C_36_H_38_O	1.28	4
	**Guantiva, Iguaque and Guerrero**				
9	515.119	8.1	1,5-di-*O*-(*E*)-dicaffeoylquinic acid	3.05	1
22	353.088	4.5	5-*O*-(*E*)-caffeoylquinic acid	2.73	1
23	329.234	12.6	C_22_H_34_O_2_	2.10	4
27	695.124	9.7	2,3,5 or 2,4,5-tricaffeoylaltraric acid	2.04	1
38	515.119	5.8	1,3-di-*O*-(*E*)-dicaffeoylquinic acid	1.66	1
45	515.119	8.6	3,4-di-*O*-(*E*)-dicaffeoylquinic acid	1.49	1
	**Chingaza and Cruz Verde**				
1	315.051	11.1	3-*O*-methylquercetin	7.11	1
7	209.029	0.9	altraric acid	4.24	2
2	631.109	11.1	dimer of 3-*O*-methylquercetin	3.74	1
26	153.018	2.8	protocatechuic acid	3.39	1
6	299.020	15.0	fragment of biflavonoid (C_31_H_20_O_14_)	3.38	3
10	615.079	15.0	biflavonoid (C_31_H_20_O_14_)	3.03	3
17	301.035	10.7	quercetin	1.98	1
	**Rabanal**				
3	195.050	0.9	gluconic acid or isomers	8.90	3
18	179.055	0.9	hexose	2.76	3

* Confidence level achieved in the identification of metabolites: 1 (high), identified by Rt and accurate MS comparisons with a reference standard; 2 (intermediate), identified by accurate MS comparisons and online searches in the Dictionary of Natural Products and AsterBioChem databases and by interpretation of fragmentation patterns; 3 (low), chemical class suggested by accurate MS comparisons with online databases and chemotaxonomy information; 4 (lowest), unknown metabolites [49]. Discriminant metabolites were selected based on their VIP scores > 1.0 and contribution in the loadings plot (first variables with a value higher than 0.7).

**Table 4:** Non-metric multidimensional scaling (NMDS) model statistics obtained by the correlation between the metabolic fingerprints of 109 taxa of Espeletiinae (representing 4181 variables) and their locality of collection, taxonomy, and climate as supplementary variables. Variables with a *p*-value < 0.01 highlighted in bold.

Variable	NMDS1	NMDS2	R^2^	Pr(>r)
**Locality**	0.9417	0.3364	0.5295	**9.9 × 10^−5^**
**Genus**	0.9932	0.1167	0.2982	**9.9 × 10^−5^**
**Temperature**	0.9249	0.3803	0.2072	**9.9 × 10^−5^**
**Elevation**	−0.9964	−0.0843	0.1391	**4.0 × 10^−4^**
Solar radiation	−0.2752	−0.9614	0.0814	0.0117
Season	0.5502	0.8350	0.0459	0.0853
Rainfall	0.7026	0.7116	0.0253	0.2505

NMDS1 and NMDS2 account for the two reduced dimensions considered in the analysis. R^2^ (coefficient of determination) represents the proportion of variance in the dependent variable that is predictable from the independent variables.

**Table 5:** Number of samples of Espeletiinae analyzed in the present study.

Genus	Determined to Species	Determined to Genus	Replicates	Total Samples
*Espeletia*	64	28	86	178
*Espeletiopsis*	4	0	10	14
*Liban* *othamnus*	?	8	2	10
*Ruilopezia*	?	6	0	6
*Paramiflos*	1	0	1	2
**Total**	≥71	42	99	210

## Data Availability

Raw chromatographic data and final datasets supporting the metabolomics experiments are publicly available in two online repositories: MetaboLights (study identifiers: MTBLS943 and MTBLS944, https://www.ebi.ac.uk/metabolights/) and MassIVE (study identifier MSV000085122, https://massive.ucsd.edu/ProteoSAFe/static/massive.jsp). R codes are available in the Supplementary Materials.

## Supplementary Materials

The following are available online at https://www.mdpi.com/article/10.3390/metabo11040220/s1. Compounds annotations by interpretation of fragmentation patterns; Figure S1: Richness of Espeletiinae species in the Andes of Colombia, Ecuador, and Venezuela, Figure S2: Superimposed base peak chromatograms of Colombian and Venezuelan samples of Espeletiinae showing the discriminant metabolites associated with the sample’s country of origin, Figure S3: Non-metric multidimensional scaling (NMDS) showing the correlation between the metabolic fingerprints of 109 taxa of Colombian (A) and Venezuelan (B) Espeletiinae, Figure S4: PCA scores plot resulting from the analysis of negative ion mode dataset with variables transformed to binary values, Figure S5: Decision-tree resulting from the analysis of negative ion mode dataset with variables transformed to binary values, Table S1: Detailed mass spectrometry information of all dereplicated metabolites in species of Espeletiinae, Table S2: Names, codes, geographical origin and environmental variables associated with the investigated samples of Espeletiinae, Table S3: Names, codes, and geographical origin of the samples of *Espeletia* investigated in the intraspecific dataset, Table S4: Model statistics for the interspecific and intraspecific datasets.
