# Supplementary material for: Effect of the Andean Geography and Climate on the Specialized Metabolism of Its Vegetation: The Subtribe Espeletiinae (Asteraceae) as a Case Example
Source: Metabolites. 2021 Apr 4;11(4):220. doi: 10.3390/metabo11040220 (PMC8065660; doi:10.3390/metabo11040220)
Supplement: Supplementary file 1 [file metabolites-11-00220-s001.pdf]

metabolites

Supplementary Materials

Effect of the Andean Geography and Climate on the Specialized Metabolism of its Vegetation: The Subtribe Espeletiinae (Asteraceae) as a Case Example

Guillermo F. Padilla-González[1,2], Mauricio Diazgranados[3] and Fernando B. Da Costa[1,*]

[1] AsterBioChem Research Team, Laboratory of Pharmacognosy, School of Pharmaceutical Sciences of Ribeirão Preto, University of São Paulo, Ribeirão Preto, SP, Brazil
[2] Jodrell Laboratory, Royal Botanic Gardens, Kew, Kew Road, London TW9 3AB, United Kingdom; f.padilla@kew.org
[3] Millennium Seed Bank, Royal Botanic Gardens, Kew, Ardingly, West Sussex, United Kingdom; m.diazgranados@kew.org
* Correspondence: febcosta@fcfrp.usp.br

Supplementary Materials

Compounds annotations:

Free organic acids

Seven compounds belonging to the class of free organic acids were tentatively annotated based on accurate mass comparisons with reference standards analyzed in identical experimental conditions and by online database searches. Compounds 3 and 8 (Table S1) were annotated by retention time and accurate mass comparisons with references standards. Compound 3, annotated as quinic acid, showed a parent ion at $[M-H]^-$ 191.05534 *m/z* and a retention time of 0.98 min. The pure standard of quinic acid eluted in a retention time of 1.01 min and showed a parent ion at $[M-H]^-$ 191.05515 *m/z.* Compound 8 was annotated as protocatechuic acid. This compound showed a parent ion at $[M-H]^-$ 153.01825 *m/z*, with a similar *m/z* value for the pure standard ($[M-H]^-$ 153.01796 *m/z*). Compound 1 (Table S1) was proposed as gluconic acid or its isomers. This compound produced a parent ion at $[M-H]^-$ 195.05013 *m/z* and database searches in the Dictionary of Natural Products (DNP) showed twelve possible candidates with a molecular formula of $C_6H_{12}O_7$, all of them gluconic acid isomers which are produced from glucose [1]. A similar approach was followed for compound 4, proposed as xylonic acid or its isomers, and for compound 7, proposed as malic acid or methoxydroxypropanedioic acid (Table S1). Compound 4 showed a parent ion at $[M-H]^-$ 165.03951 *m/z,* consistent with the formula $C_5H_{10}O_6$. Accurate mass searches in the DNP showed five possible candidates, four of them corresponding to the different configurations of xylonic acid and one of them to its isomer apionic acid. Compound 7, proposed as malic acid or its isomers, displayed a parent ion at $[M-H]^-$ 133.01317 *m/z*, consistent with the formula $C_4H_6O_5$. DNP searches showed six possible candidates, five of them corresponding to malic acid and its isomers and the remaining one to methoxydroxypropanedioic acid. Compound 6 was proposed as acetylglucuronic acid based on database searches in the DNP and literature information. This compound, previously reported in *Smallanthus sonchifolius* [2], a species belonging to the sister genus of Espeletiinae, showed a parent ion at $[M-H]^-$ 235.04552 *m/z* and DNP searches afforded six possible candidates, all of them isomers of acetylglucuronic acid. Lastly, compound 5 was annotated as altraric acid (Table S1). This compound displayed a parent ion at $[M-H]^-$ 209.02950 *m/z* and two fragment ions at 191.01929bp *m/z* (representing a neutral loss of H_2O) and 133.01329 *m/z* (representing a neutral loss of $C_2H_4O_3$), which is in accordance with previous literature reports [3]. Altraric acid has been

previously reported in species of Espeletiinae and its sister genus *Smallanthus* [2,4]. Excluding compounds 3 and 8, identified by comparisons with reference standards (level 1, Metabolomics Standards Initiative – MSI) and compound 5, identified by database comparisons, chemotaxonomy and MS/MS data (level 2, MSI), the annotation of the remaining organic acids is still highly tentative, and they correspond to level 3 according to the MSI [5].

Caffeic acid derivatives

Eight caffeic acid derivatives were annotated based on comparisons with pure standards (6 compounds) and MS/MS data (2 compounds). Compounds 9, 10, 12-14 and 16 (Table S1) were identified by retention time, accurate mass values and MS/MS comparisons with pure standards. Compound 9 (Rt = 4.52 min), identified as 5-*O*-(*E*)-caffeoylquinic acid, displayed a parent ion at $[M-H]^-$ 353.08780 *m/z* and a fragment ion at 191.05539 *m/z* consistent with a neutral loss of a caffeoyl unit. Although analysis of pure standards of 3-*O*-(*E*)-caffeoylquinic acid and 5-*O*-(*E*)-caffeoylquinic acid showed both similar mass values and fragment ions to compound 9 (different from the other two chlorogenic acid isomers at positions 1 and 4), there was a difference of more than one minute in the retention time value in the standards of these two isomers (3.22 min for 3-*O*-(*E*)-caffeoylquinic acid and 4.45 min for 5-*O*-(*E*)-caffeoylquinic), suggesting that compound 9 corresponds to 5-*O*-(*E*)-caffeoylquinic acid. Compounds 10, 12, 13 and 14 (Table S1) were all identified as different dicaffeoylquinic acid isomers based on Rt and HRMS data comparisons with pure standards. According to Clifford et al. (2005) significant differences in the retention time and MS^2 fragment intensity among dicaffeoylquinic acid isomers allow their unambiguous characterization even if pure standards are not available [6]. For example, 4-acyl dicaffeoylquinic acids are easily distinguished by the presence of a base peak at 173 *m/z* in their MS^2 spectrum, while the isomers 1,3-, 1,5- and 3,5-*O*-(*E*)-dicaffeoylquinic acids can be distinguished by the relative proportion of the fragment ions at 335 *m/z* and 179 *m/z*. Compound 16, annotated as 2,3,5 or 2,4,5-tricaffeoylaltraric acid, was also identified by Rt and HRMS comparisons with a pure standard (Table S1), previously isolated in our laboratory from the roots of *Smallanthus sonchifolius* [7,8]. Although the structure of this compound was previously elucidated based on comprehensive uni- and bidimensional NMR data [7], due to the symmetry of the altraric acid core molecule, resolving the ambiguity in the esterification of the caffeic acid molecule at position C3 and C4 is especially difficult, as previously described by [9]. Therefore, the identity of this compound in the literature remains relative as 2,3,5 or 2,4,5-tricaffeoylaltraric acid. The HRMS spectrum of compound 16 showed a parent ion at $[M-H]^-$ 695.12439 *m/z* and fragment ions at 533.09344 *m/z*, 371.06146 *m/z*, 209.02939bp *m/z*, consistent with consecutive neutral losses of three caffeoyl units. The same accurate mass and fragmentation pattern was observed for compound 15 (Table S1). However, considering its retention time difference of 0.6 min relative to compound 16, it was proposed as another tricaffeoylaltraric acid isomer. Lastly, compound 11 (Table S1) was proposed as methyl 3-O-caffeoyl-4-O-feruloylquinate (Rt = 8.01 min) based on MS/MS data and previous literature reports for species of the genus *Espeletia* [4]. This compound showed a precursor ion at 543.15106 *m/z* in the negative ionization mode and 545.16528 *m/z* in the positive mode. In this last ionization mode, the molecule showed extensive fragmentation in the ionization source, showing peaks at 527.15454 *m/z*, 351.10733 *m/z* consistent with a neutral loss of a water molecule in the first case and a feruloyl unit in the second. Two additional fragment peaks were observed at 177.05457 *m/z* and 163.03885 *m/z* originated after the loss of a caffeoylquinate and a feruloylquinate unit, respectively.

Flavonoids

The tentative identity of three flavonoids (Compounds 17, 22 and 23, Table S1) was proposed based on Rt, accurate mass and MS/MS comparisons with online databases and literature information. Two additional compounds (Compounds 18 and 19, Table S1) were unambiguously identified as quercetin and 3-*O*-methylquercetin, respectively, by Rt and MS comparisons with pure standards. Both compounds have been previously isolated from species of the genus *Espeletia* [10] and appear to be widely distributed in the subtribe Espeletiinae [4]. Compound 22 was annotated as di-*O*-methylquercetin based on database searches and interpretation of its fragmentation pattern (Table S1). This compound showed a parent ion at $[M-H]^-$ 329.06683 *m/z* and a similar fragmentation pattern to 3-*O*-methylquercetin, but it was characterized by two consecutive losses of 15 Da, consistent with two methyl radicals to produce fragment ions at 314.04343 *m/z* and 299.01987

m/z. Compound 17, annotated as quercetin-3-*O*-cinnamoyl-hexoside (Table S1), showed a parent ion at $[M-H]^-$ 609.12524 *m/z* and a fragment ion at 463.08878 *m/z* resulting from the neutral loss of 146.036 Da, consistent with a cinnamoyl unit. The presence of two additional fragments at 301.03455 *m/z* and 300.02771 *m/z* suggest not only a neutral loss of 162.054 Da (consistent with a hexose unit) but also the presence of quercetin as the aglycone backbone esterified at position 3 of ring C. The presence of additional fragments at 271.02521 *m/z* and 151.00272 *m/z* provide further support for the presence of quercetin as flavonoid aglycone. Compound 21 (Table S1), was assigned as unknown as no conclusive results were obtained from the analysis of its mass spectrum. This compound showed a parent ion at $[M-H]^-$ 301.07178 *m/z* consistent with the molecular formula $C_{16}H_{14}O_6$ and fragment ions at 299.02011 *m/z*, 165.01848 *m/z* and 135.04407 *m/z*. Database searches in the DNP afforded 283 library hits from different chemical classes.. Lastly, the tentative presence of a biflavonoid with a molecular formula of $C_{31}H_{20}O_{14}$ was proposed by online database searches and interpretation of its fragmentation pattern. This compound showed a parent ion at $[M-H]^-$ 615.07874 *m/z*, a dimer at 1231.163 *m/z*, and an *in*-source fragment ion at 299.01974 *m/z*, representing a neutral loss of 316.058 Da, consistent with the accurate mass for a methoxylated quercetin unit. The presence of additional fragment ions at 271.02509 *m/z* and 243.03000 *m/z*, which characterize flavonols, suggest this metabolite is likely a biflavonoid with methoxyquercetin as one of the flavonoid units. Database searches in Scifinder and DNP using the calculated molecular formula ($C_{31}H_{20}O_{14}$) showed only three potential candidate structures, one of which was a flavonoid (8,8''-methylene-bisquercetin) previously described in Asteraceae [11]. Although the accurate mass of 8,8''-methylene-bisquercetin is consistent with our experimental results, the fragmentation pattern of this biflavonoid does not show the neutral loss of 316.058 Da observed in the present study or the loss of 15 Da representing a methyl radical, suggesting our molecule is not 8,8''-methylene-bisquercetin and might correspond to a potentially new metabolite. However, the isolation and structural elucidation of this metabolite is still necessary for more robust conclusions about its chemical structure.

Diterpenes and triterpenes

The identity of two diterpenes and five triterpenes was tentatively proposed based on accurate mass comparisons with metabolites previously reported in the genus *Espeletia* and by online database searches. Compound 25 (Table S1) was proposed as hydroxy-*ent*-kauren-18-oic acid. This metabolite showed a parent ion at $[M-H]^-$ 317.21246 *m/z* and a fragment ion at 287.20209 *m/z*, consistent with a neutral loss of CH_2O. This compound has been previously reported to occur in the subtribe Espeletiinae [12]. The presence of an additional diterpene (compound 24) with a parent ion at $[M-H]^-$ 333.20718 *m/z* was also suggested based on DNP searches and the widespread occurrence of this chemical class in Espeletiinae [13].. Lastly, the tentative identity of one additional compound (Compound 20, Table S1) belonging to the class of antraquinones was proposed based on accurate mass comparisons and database searches. This metabolite, proposed as 5-hydroxyanthraquinone-1,3-dicarboxylic acid, showed a parent ion at $[M-H]^-$ 311.02036 *m/z*. Database searches in the DNP showed only one potential match with the same accurate mass value.

References

1. Kim, H.Y.; Park, H.M.; Lee, C.H. Mass spectrometry-based chemotaxonomic classification of *Penicillium* species (*P. echinulatum*, *P. expansum*, *P. solitum*, and *P. oxalicum*) and its correlation with antioxidant activity. *J. Microbiol. Methods* **2012**, *90*, 327–335, doi:10.1016/j.mimet.2012.06.006.

2. Padilla-González, G.F.; Frey, M.; Gómez-Zeledón, J.; Da Costa, F.B.; Spring, O. Metabolomic and gene expression approaches reveal the developmental and environmental regulation of the secondary metabolism of yacón (*Smallanthus sonchifolius*, Asteraceae). *Sci. Rep.* **2019**, *9*, 13178, doi:10.1038/s41598-019-49246-2.

3. Žiarovská, J.; Padilla-González, G.F.F.; Viehmannová, I.; Fernández, E. Genetic and chemical diversity among yacon [*Smallanthus sonchifolius* (Poepp. et Endl.) H. Robinson] accessions based on iPBS markers and metabolomic fingerprinting. *Plant Physiol. Biochem.* **2019**, *141*, 183–192, doi:10.1016/j.plaphy.2019.05.020.

4. Padilla-González, G.F.; Diazgranados, M.; Da Costa, F.B. Biogeography shaped the metabolome of the genus *Espeletia*: A phytochemical perspective on an Andean adaptive radiation. *Sci. Rep.* **2017**, *7*, 8835, doi:10.1038/s41598-017-09431-7.

5. Sumner, L.W.; Amberg, A.; Barrett, D.; Beale, M.H.; Beger, R.; Daykin, C.A.; Fan, T.W.-M.; Fiehn, O.; Goodacre, R.; Griffin, J.L.; et al. Proposed minimum reporting standards for chemical analysis. *Metabolomics* **2007**, *3*, 211–221, doi:10.1007/s11306-007-0082-2.

6. Clifford, M.N.; Knight, S.; Kuhnert, N. Discriminating between the six isomers of dicaffeoylquinic acid by LC-MSn. *J. Agric. Food Chem.* **2005**, *53*, 3821–3832, doi:10.1021/jf050046h.

7. Padilla-González, G.F.; Sadgrove, N.J.; Ccana-Ccapatinta, G.V.; Leuner, O.; Fernandez-Cusimamani, E. Feature-based molecular networking to target the isolation of new caffeic acid esters from yacon (*Smallanthus sonchifolius*, Asteraceae). *Metabolites* **2020**, *10*, doi:10.3390/metabo10100407.

8. Padilla-González, G.F.; Amrehn, E.; Frey, M.; Gómez-Zeledón, J.; Kaa, A.; Da Costa, F.B.; Spring, O. Metabolomic and gene expression studies reveal the diversity, distribution and spatial regulation of the specialized metabolism of yacón (*Smallanthus sonchifolius*, Asteraceae). *Int. J. Mol. Sci.* **2020**, *21*, 4555, doi:10.3390/ijms21124555.

9. Takenaka, M.; Yan, X.; Ono, H.; Yoshida, M.; Nagata, T.; Nakanishi, T. Caffeic acid derivatives in the roots of yacon (*Smallanthus sonchifolius*). *J. Agric. Food Chem.* **2003**, *51*, 793–796, doi:10.1021/jf020735i.

10. Padilla-Gonzalez, G.F.; Diazgranados, M.; Ccana-Ccapatinta, G.; Casoti, R.; Da Costa, F.B. Caffeic acid derivatives and further compounds from *Espeletia barclayana* Cuatrec. (Asteraceae, Espeletiinae). *Biochem. Syst. Ecol.* **2017**, *70*, 291–293, doi:10.1016/j.bse.2016.12.016.

11. Martucci, M.E.P.; De Vos, R.C.H.; Carollo, C.A.; Gobbo-Neto, L. Metabolomics as a potential chemotaxonomical tool: Application in the genus *Vernonia* schreb. *PLoS One* **2014**, *9*, e93149, doi:10.1371/journal.pone.0093149.

12. Bohlmann, F.; Suding, H.; Cuatrecasas, J.; King, R.M.; Robinson, H. New diterpenes from subtribe Espeletiinae. *Phytochemistry* **1980**, *19*, 267–271, doi:10.1016/s0031-9422(00)81971-8.

13. Usubillaga, A.; de Hernandez, J.; Perez, N.; Kiriakidis, M. Kauranoid diterpenes in Espletia species. *Phytochemistry* **1973**, *12*, 2999, doi:10.1016/0031-9422(73)80526-6.

metabolites

MDPI

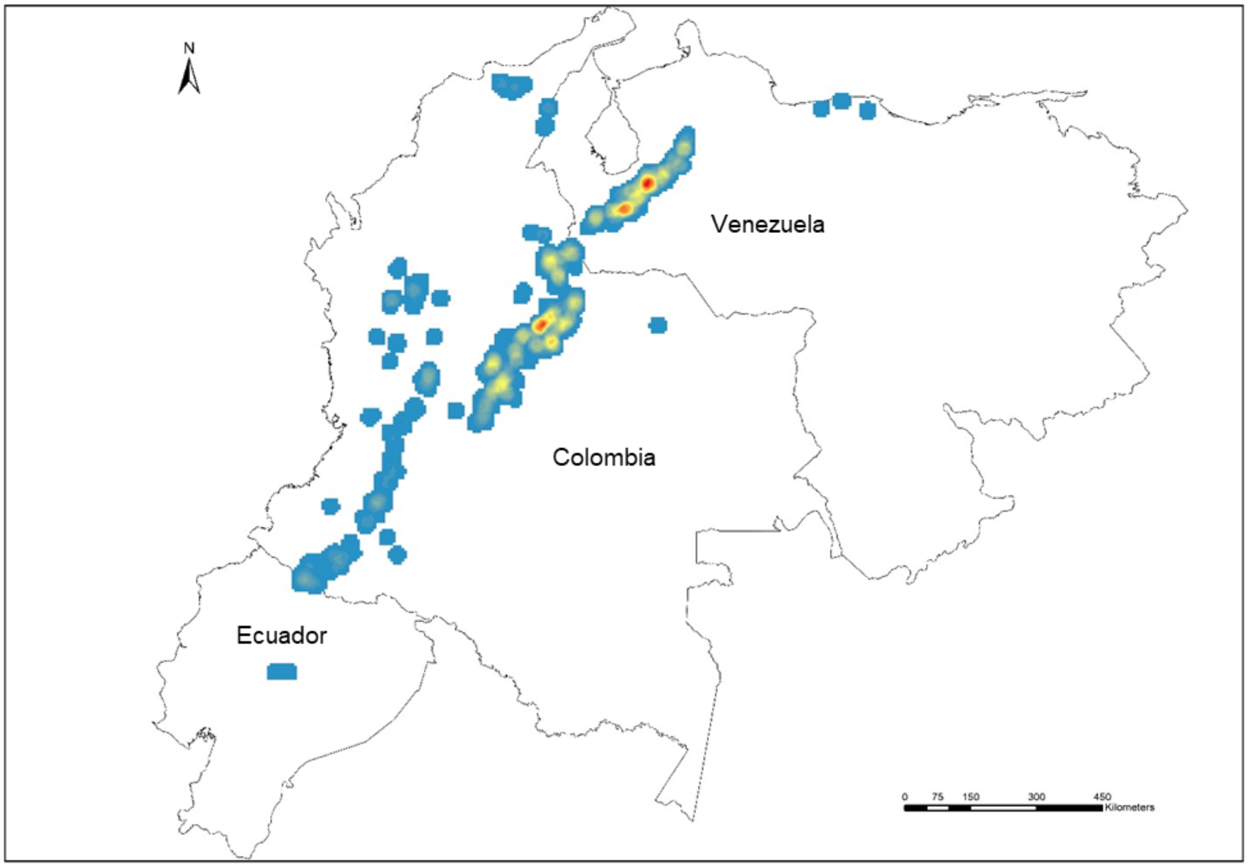

Figure S1. Richness of Espeletiinae species in the Andes of Colombia, Ecuador and Venezuela. Dark yellow and red spots represent the areas with the highest number of species of Espeletiinae. Map created in the software ArcGIS 10.7.

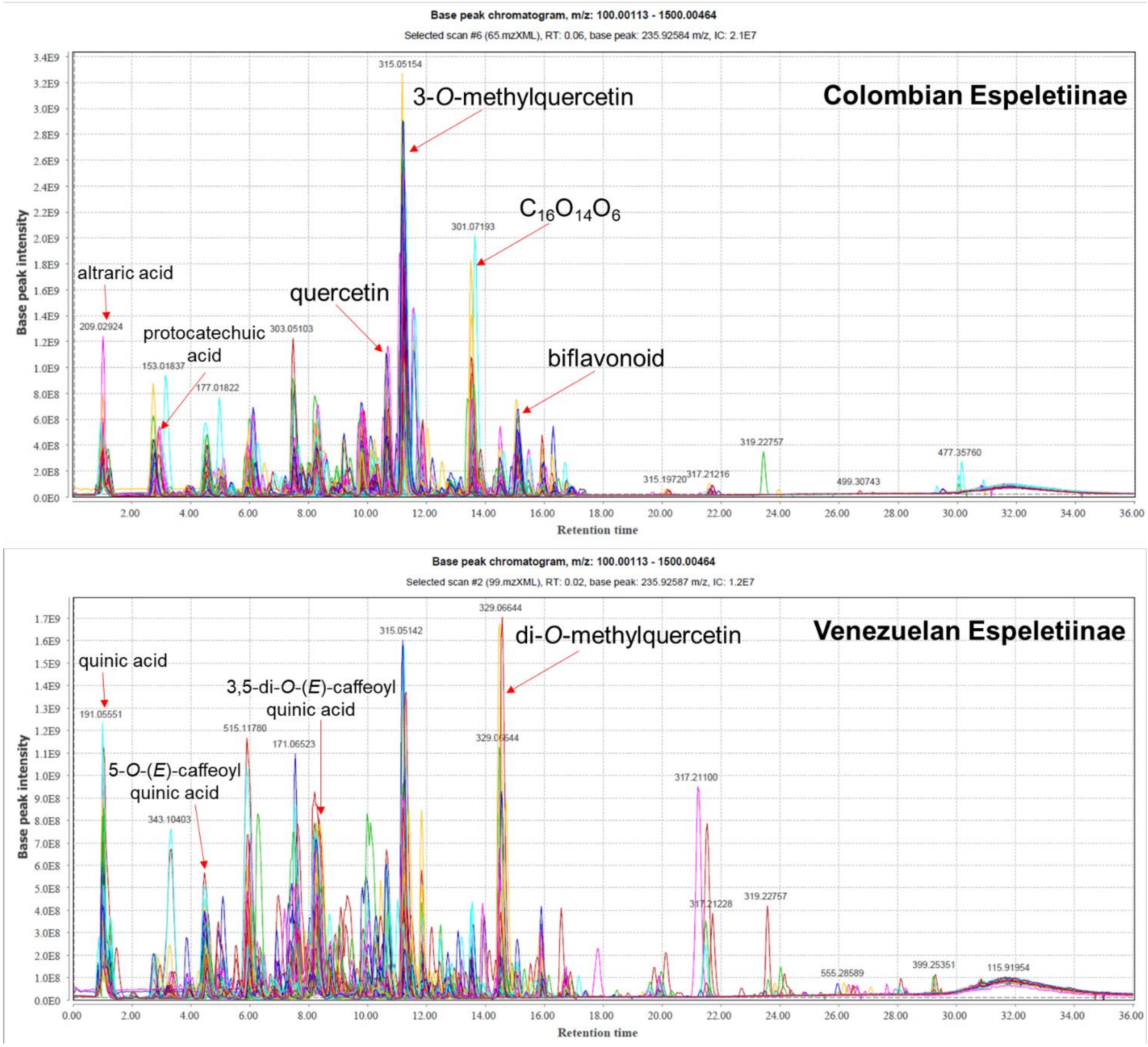

Figure S2. Superimposed base peak chromatograms of Colombian and Venezuelan samples of Espeletiinae showing the discriminant metabolites associated with the sample's country of origin.

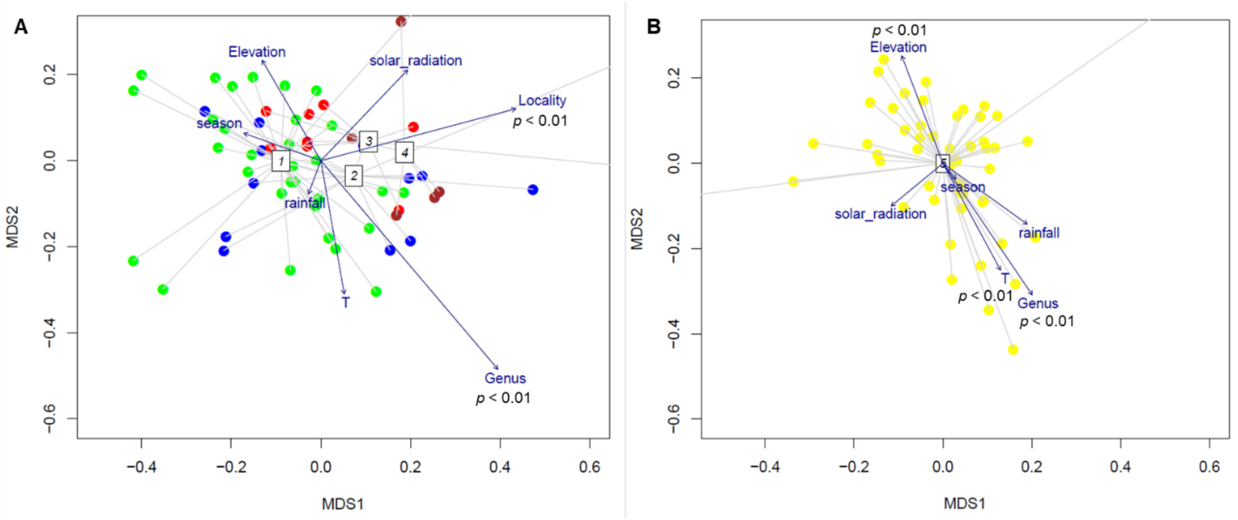

Figure S3. Non-metric multidimensional scaling (NMDS) showing the correlation between the metabolic fingerprints of 109 taxa of Colombian (**A**) and Venezuelan (**B**) Espeletiinae obtained by UHPLC-HRMS in negative ion mode and their geographic localities, taxonomic genus, elevation, and environmental variables.

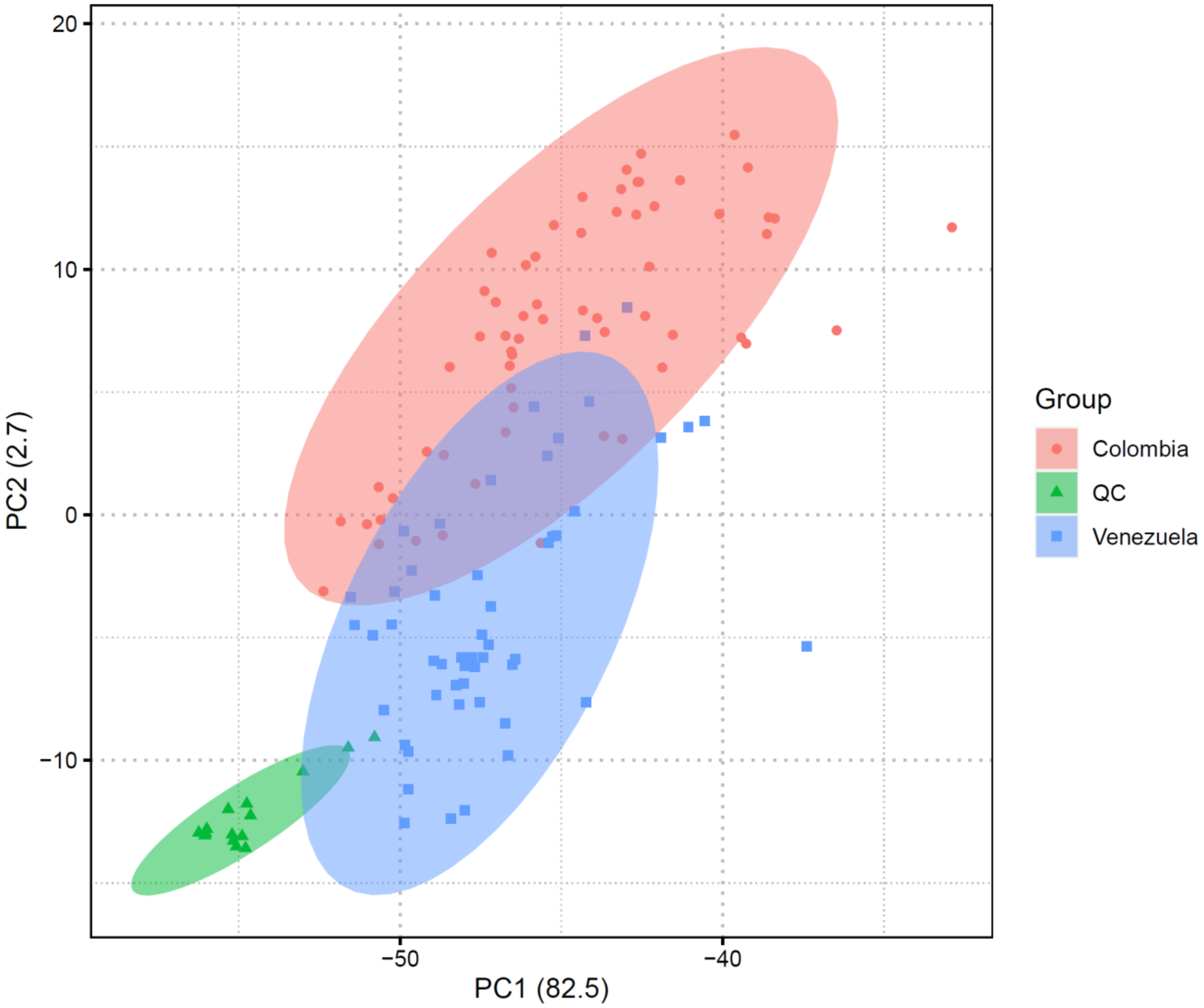

Figure S4. PCA scores plot resulting from the analysis of negative ion mode dataset with variables transformed to binary values.

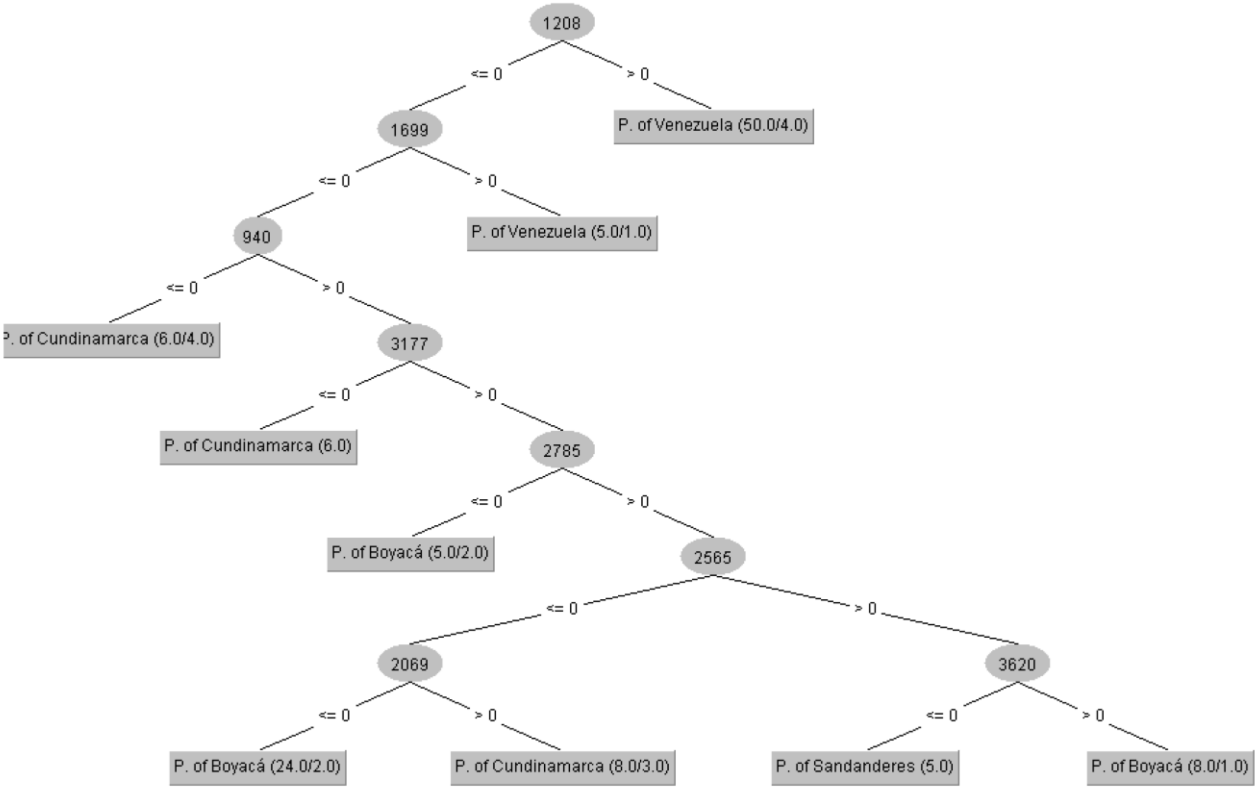

Figure S5. Decision-tree resulting from the analysis of negative ion mode dataset with variables transformed to binary values.

metabolites

MDPI

Table S1. Detailed mass spectrometry information of all dereplicated metabolites in species of Espeletiinae.

No	Rt (min)	Tentative compound	Negative ionization ESI Deprotonated molecule / in-source fragments	Negative MS/MS (AIF mode)	Positive ionization ESI Protonated molecule / in-source fragments	Confidence level*
1	0.9	gluconic acid or isomers	$[M - H]^-$ 195.05013	-	-	3
2	0.9	hexose	$[M - H]^-$ 179.05501	-	-	3
3	1.0	quinic acid	$[M - H]^-$ 191.05534	-	-	1
4	1.0	xylonic acid or isomers	$[M - H]^-$ 165.03951	-	-	3
5	0.9	altraric acid	$[M - H]^-$ 209.02950	191.01929bp, 133.01329	-	2
6	1.0	acetylglucuronic acid or isomers	$[M - H]^-$ 235.04552	-	-	3
7	1.1	malic acid or methoxypropanedioic acid	$[M - H]^-$ 133.01317	-	-	3
8	2.8	protocatechuic acid	$[M - H]^-$ 153.01825	-	-	1
9	4.5	5-*O*-(*E*)-caffeoylquinic acid	$[M - H]^-$ 353.08780	191.05539bp	$[M + H]^+$ 355.10226 / $[(M + H) - QA]^+$ 163.03893	1

10	5.8	1,3-*O*-(*E*)-dicaffeoylquinic acid	$[M - H]^-$ 515.11902	353.08771, 335.07791, 191.05518bp, 179.03401, 135.04395	$[M + H]^+$ 517.13336	1
11	8.0	methyl 3-*O*-caffeoyl-4-*O*-feruloylquinate	$[M - H]^-$ 543.15106	-	$[M + H]^+$ 545.16528 / $[(M + H) - H_2O]^+$ 527.15454, $[(M + H) - C_7H_8O_2]^+$ 421.11270, $[(M + H) - H_2O - FER]^+$ 351.10733, $[(M + H) - FER - 2(H_2O)]^+$ 333.08618 $[(M + H) - C_{17}H_{19}O_9]^+$ 177.05457, $[(M + H) - C_{18}H_{21}O_9]^+$ 163.03885	2
12	8.1	1,5-*O*-(*E*)-dicaffeoylquinic acid	$[M - H]^-$ 515.11963	353.08820, 335.07724, 191.05542bp, 179.03429, 135.04411	$[M + H]^+$ 517.13428	1
13	8.4	3,5-*O*-(*E*)-dicaffeoylquinic acid	$[M - H]^-$ 515.11908	353.08762, 191.05511bp, 179.03401, 135.04395	$[M + H]^+$ 517.13342	1
14	8.6	3,4-*O*-(*E*)-dicaffeoylquinic acid	$[M - H]^-$ 515.11945	353.08801, 191.05534, 179.03409, 173.04471bp, 135.04404	$[M + H]^+$ 517.13422	1
15	9.1	tricaffeoylaltraric acid isomer	$[M - H]^-$ 695.12476	533.09357, 371.06177, 209.02945bp	-	2
16	9.7	2,3,5 or 2,4,5-tricaffeoylaltraric acid	$[M - H]^-$ 695.12439	533.09344, 371.06146, 209.02939bp	$[M + H]^+$ 697.13916	1

17	9.9	quercetin-3-*O*-cinnamoyl-hexoside	$[M - H]^-$ 609.12524	463.08878bp, 301.03455, 300.02771, 271.02521, 151.00272	$[M + H]^+$ 611.13934 / $[(M + H) – \text{cinnamoyl hexoside}]^+$ 303.04944	2
18	10.7	quercetin	$[M - H]^-$ 301.03506	271.02487, 255.02972, 243.02966, 151.00256bp	$[M + H]^+$ 303.04984	1
19	11.1	3-*O*-methylquercetin	$[M - H]^-$ 315.05087	300.02701, 271.02451bp, 255.02948, 243.02948	$[M + H]^+$ 317.06461	1
20	12.6	5-hydroxyanthraquinone-1,3-dicarboxylic acid	$[M - H]^-$ 311.02036	-	$[M + H]^+$ 313.03442	3
21	13.6	$C_{16}H_{14}O_6$	$[M - H]^-$ 301.07178	299.02011, 165.01848, 135.04407bp	$[M + H]^+$ 303.08633	4
22	14.5	di-*O*-methylquercetin	$[M - H]^-$ 329.06683	314.04343, 299.01987bp, 271.02496, 243.02988	$[M + H]^+$ 331.08075	2
23	15.0	biflavonoid ($C_{31}H_{20}O_{14}$)	$[M - H]^-$ 615.07874 / $[(M - H) - QER - CH_3]^-$ 299.01990	299.01974bp, 271.02509, 243.03000	$[M + H]^+$ 617.09271	3
24	16.1	putative diterpene ($C_{20}H_{30}O_4$)	$[M - H]^-$ 333.20718	-	$[M + H]^+$ 335.22122 / $[(M + H) - H_2O]^+$ 317.21103, $[(M + H) - 2xH_2O]^+$ 299.20050	3
25	22.0	hydroxy-*ent*-kauren-18-oic acid	$[M - H]^-$ 317.21246	287.20209	$[M + H]^+$ 319.22742	3
26	28.1	$C_{36}H_{38}O$	$[M - H]^-$ 485.28284	-	-	4

27	31.5	$C_{36}H_{38}O$	$[M - H]^-$ 485.28284	-	-	4
28	32.5	$C_{36}H_{38}O$	$[M - H]^-$ 485.28284	-	-	4
29	34.2	$C_{36}H_{38}O$	$[M - H]^-$ 485.28265	-	-	4
30	35.4	$C_{36}H_{38}O$	$[M - H]^-$ 485.28284	-	-	4

* Confidence level achieved in the identification of metabolites: 1 (high), identified by Rt and accurate MS comparisons with a reference standard; 2 (intermediate), identified by accurate MS comparisons and online searches in the Dictionary of Natural Products and AsterBioChem databases and by interpretation of fragmentation patterns; 3 (low), chemical class suggested by accurate MS comparisons with online databases and chemotaxonomy information; 4 (lowest), unknown metabolites [5]. CAF: caffeoyl moiety; bp: base peak; QER: quercetin; FER: feruloyl moiety; QA: quinic acid. ESI-HRMS (resolution of 70,000 FWHM) and ESI-HCD MS/MS (resolution of 35,000 FWHM) spectra were obtained for the positive (voltage = +3.6 kV) and negative (voltage = −3.2 kV) ionization modes using the *Full scan* and AIF mode with a normalized collision energy 35 eV.

metabolites

MDPI

Table S4. Model statistics for the interspecific and intraspecific datasets.

Dataset	PC	Explained variance (%)	Eigenvalue	R^2 (OPLS-DA)	Q^2 (OPLS-DA)
Interspecific	1	14.1	15.9		
	2	7.0	7.97		
	3	5.9	4.82		
Intraspecific	1	29.3	7.33	0.96	0.77
	2	15.1	3.77		
	3	10.2	2.55		
E. argentea	1	38.2	3.44	0.99	0.93
	2	29.6	2.66		
	3	12.6	-		
E. boyacensis	1	48.5	2.91	1.00	0.99
	2	22.5	1.35		
	3	14.5	-		
E. grandiflora	1	31.7	3.17	1.00	0.94
	2	21.7	2.17		
	3	15.0	-		

R code used for NMDS analysis

```
teste <- read.csv("Espeletiinae.neg.csv", h=T)

rownames(teste) <- teste[,1]

teste <- teste[,3:4183]

teste.env <- read.csv("Espeletiinae.enviro.nmds(log).csv", h=T)

rownames(teste.env) <- teste.env[,1]

teste.env <- teste.env[,2:7]

library(vegan)

nmds.teste <- metaMDS(teste, wascores=TRUE, k=3, distance="bray",
trymax=100)

nmds.teste.env <- metaMDS(teste.env, wascores=TRUE, k=3,
distance="bray")

stressplot(nmds.teste)

nmds.envfit <- envfit(nmds.teste, teste.env, permu=10000)
```

```
co=c("green", "blue", "red", "brown", "yellow")

shape=c(16, 16, 16, 16, 16)

plot(nmds.teste$points, col=co[teste.env$Locality], pch =
shape[teste.env$Locality], xlim=c(-0.5,0.6), ylim=c(-0.61,0.3),
cex=1.5)

ordispider(nmds.teste, groups = teste.env$Locality,
col="lightgray", label = TRUE, cex=0.8, font=c(3))

plot(nmds.envfit, cex=0.9, p.max=1, col="darkblue")

print(nmds.envfit)
```
